# Genomic Variation Underlying the Breeding Selection of Quinoa Varieties Longli-4 and CA3-1 in China

**DOI:** 10.3390/ijms232214030

**Published:** 2022-11-14

**Authors:** Xiaofeng Li, Ruilan Ran, Guoxiong Chen, Pengshan Zhao

**Affiliations:** 1 Key Laboratory of Stress Physiology and Ecology in Cold and Arid Regions, Gansu Province, Northwest Institute of Eco-Environment and Resources, Chinese Academy of Sciences, Lanzhou 730000, China; 2 University of Chinese Academy of Sciences, Beijing 100049, China; 3 Shapotou Desert Research & Experiment Station, Northwest Institute of Eco-Environment and Resources, Chinese Academy of Sciences, Lanzhou 730000, China

**Keywords:** quinoa, genetic variation, conventional breeding, heterozygosity

## Abstract

Quinoa (*Chenopodium quinoa*) is a well-known climate-resilient crop and has been introduced into multiple marginal lands across the world, including China, to improve food security and/or balanced nutrient supplies. Conventional breeding has been widely applied in the selection and breeding of quinoa varieties in China since 1980s; however, few studies have been implemented on the genetic variances among different varieties developed by diversity breeding objectives. In this study, the phenotypic and genetic differences between two varieties (Longli-4 and CA3-1) from China were systematically analyzed. A total of 407,651 and 2,731,411 single nucleotide polymorphisms (SNPs) and 212,724 and 587,935 small insertion and deletion (INDELs) were detected for Longli-4 and CA3-1, respectively, when compared with the reference genome of PI614886. The SNPs/INDELs were unevenly distributed across each chromosome for both varieties. There were 143,996 SNPs and 83,410 INDELs shared between Longli-4 and CA3-1, accounting for 4% of the total variances. The variation was then screened based on the SNP effects. There were 818 and 73 genes with the variety-specific non-synonymous and stop-gain variation in Longli-4, whereas there were 13,701 and 733 genes in CA3-1. Specifically, 3501 genes with the non-synonymous variation and 74 genes with the stop-gain variation were found in both Longli-4 and CA3-1. These results suggest that convergent selection occurred during the different breeding processes. A set of candidate genes related to agronomic traits and domestication were further selected to detect the genetic divergence in detail in the two varieties. Only one domestication gene was identified having Longli-4-specific stop-gain variation. Twelve candidate genes related to betalain (1), flowering (4), seed size (2), domestication (1), and saponin (4) were identified having CA3-1-specific stop-gain variation. Interestingly, one seed size gene homologous of *CKX1* (*cytokinin oxidase/dehydrogenase 1*) had the stop-gain variation in both varieties. This research will therefore provide guidance for the molecular-assisted breeding in quinoa.

## 1. Introduction

Quinoa (*Chenopodium quinoa*) is an allotetraploid plant, belonging to the subfamily Chenopodioideae in Amaranthaceae [1,2]. Historical documents and archaeological evidence suggest that quinoa was already a traditional food for the native people of the Andes as early as 7000 years ago [3,4]. Due to its excellent tolerance to diverse environments and well-balanced nutritional values, quinoa has been characterized as a resilient crop [4,5,6] and has also been prized as a “whole grain” for human beings [7,8]. Since the year 2013 was named “the International Year of Quinoa” by the United Nations, the number of countries and regions cultivating quinoa have expanded substantially, reaching up to 100 in 2016 [9,10,11]. The potential of this species for future food security is widely appreciated by scientific communities and global societies [12].

Quinoa breeding could date back to the 1960s in Andean countries [13], whereas in the USA, Ecuador, Chile, and several other European countries such as Denmark, quinoa breeding was initiated in the 1980s [14,15]. Various conventionality breeding methods, such as mass- and individual-selection, cross-breeding (interspecific or intraspecific hybridization), and mutation breeding (γ-ray or ethyl methyl sulfonate mutagenesis), have been utilized for the genetic improvement of quinoa [4,16]. More than 6700 quinoa varieties have been reported across the global world [17]. Recently, quinoa breeding has been greatly advanced with the genetic map development, genome sequencing, and marker-assisted selection [4,18,19,20]. For instance, natural variation from the *triterpene saponin biosynthesis activation regulator-like 1* (*TSARL1*) can be employed for sweet grain breeding using marker-assisted selection [19,20].

Quinoa was introduced into China as early as in the 1960s by the former Institute of Crop Breeding and Cultivation, the Chinese Academy of Agricultural Sciences, but little relevant research was carried out [21]. The first cultivation experiment of quinoa in China was conducted by Tibet Agricultural and Animal Husbandry University and Tibet Academy of Agricultural and Animal Husbandry Sciences in 1987 [22]. Quinoa began to be cultivated on a large scale in China in 2008, represented by Jingle County, Shanxi Province. More than 20 provinces (regions) in China have quinoa cultivation, and of which, Gansu province in northwest China has the largest cultivation area, reaching up to 16.7 × 103 hectares (ha) in 2019 [23,24]. Consequently, at least 20 quinoa varieties adapted to a specific environment have been developed in China [23]. For instance, since 2010, Yang’s group in the Institute of Livestock, Grass and Green Agriculture, Gansu Academy of Agricultural Sciences, has launched the quinoa germplasm introduction, cultivation experiments, and breeding, and has developed some quinoa varieties, named Longli-1 to 4, with specific adaptability to arid growth conditions in Gansu province [25,26,27,28,29,30,31].

Individual selection is one of the main methodologies for quinoa breeding in China, which is highly dependent on genetic variation, natural mutation and hybridization [16,24]. The Longli-4 is a new variety developed from a Bolivia variety LUR-10 [28]. This variety has a short life cycle, high yield, abiotic stress tolerance and wide adaptation, which can be planted in arid regions in Gansu and Shanxi provinces, and the cool and cold regions in Inner Mongolia, Sichuan, Guizhou provinces as well as the east of Qinghai province [28]. The CA3-1 is obtained after two years of cultivation, and its parent line CA3 is a product of “Coles”, purchased in Australia in 2011 but grown in Bolivia and Peru. A field comparison between Longli-4 and CA3-1 has been conducted in Haiyuan dryland region of Ningxia Hui Autonomous Region, northwest of China, in 2019 [31]. Results showed that CA3-1 has a similar growth period, higher thousand-grain weight (3.64 vs. 3.05 g), and different grain color (dark vs. light white) when compared with Longli-4 [31]. The estimated yield of the CA3-1 is 6248 kg/ha, lower than that of the Longli-4 (7196.89 kg/ha) in the same field but higher than those (3341.25–4418.33 kg/ha) reported in other literatures [29,32]. Thus, both Longli-4 and CA3-1 have great yield potential and good environmental adaptability, and are elite lines for large-scale cultivation in arid regions as well as the cool and cold regions in China [31].

Morphological variation in germplasm resources is the prerequisite for individual selection. Individual lines with advanced/different and stable characteristics are the criteria for a new variety [4]. However, genetic variation is the base of individual selection. For each variety developed in China, to what extent and in which way does the breeding process impact the genome sequence? Moreover, different selection pressures are supposed to be exerted on the germplasm resources with different breeding objectives. There is a question of whether genomic regions or individual genes are selected in bias between different varieties. In addition, similar phenotypes or traits are commonly observed among different varieties. For instance, Longli-4 and CA3-1 both have a strong stem with weakness resistance, prematurity and high yield potential [31]. Is it possible that variances in orthologous genes/pathways are convergently selected in Longli-4 and CA3-1? Finally, the outcrossing rate of quinoa in different environments is in the range of 1.5–17.36% [4,33], and thus, maintaining the genetic homogeneity of the quinoa cultivars is difficult. To what extent are the homogeneity values maintained in Longli-4 and CA3-1 under different cultivation processes? With the release of quinoa genome information [19,34,35], it is possible to provide a broad perspective on the genetic variation of each variety. This manuscript focused on Longli-4 and CA3-1, and the genetic variation against the reference line PI614886 [19] was analyzed in each variety. The selection bias in the genome and individual genes was presented between Longli-4 and CA3-1. In the end, the variation patterns of some candidate genes involved in agronomic traits and domestication were reported, and the homogeneity of each variety was measured by several molecular markers.

## 2. Results

### 2.1. Phenotype Comparison between Longli-4 and CA3-1

**Figure 1:**
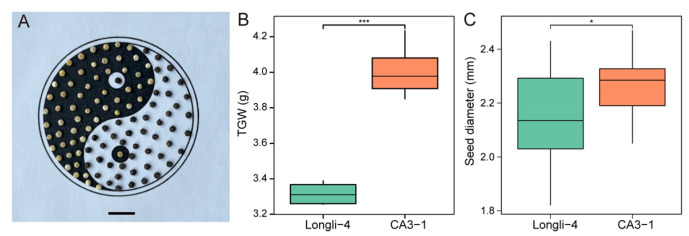
Grain size and weight of quinoa varieties. (**A**) Grain phenotype of two varieties. Longli-4 grains are light yellow and CA3-1 grains are black. Bar = 1 cm. (**B**) Thousand-grain weight (TGW) of Longli-4 and CA3-1. (**C**) Gain diameter of Longli-4 and CA3-1. *, *p* < 0.05, ***, *p* < 0.001.

Longli-4 and CA3-1 were sown in the growth chamber and a significant difference was mainly observed in mature grains (Figure 1). The colors of Longli-4 and CA3-1 grains were light-yellow and dark, respectively. The thousand-grain weight (TGW) and grain diameter of Longli-4 were significantly lower than those of CA3-1, although at different statistical levels. The stem of Longli-4 plant was in green color, while the CA3-1 was in purple (Supplemental Figure S1A). The internode lengths of the Longli-4 and CA3-1 varied differently in the top, middle, and basal stems, but the whole plant heights were similar in these two varieties (Supplemental Figure S1B–I). Likewise, no obvious difference was observed in lateral branch number, stem diameter, leaf area, panicle length, and panicle diameter (Supplemental Figure S1J–M,P–R). The top inflorescences exhibited different types within and between varieties, partly due to the variation in pedicel length, but the flowering time was similar between Longli-4 and CA3-1 (Supplemental Figure S1M), which is consistent with previous field assays [31]. The hermaphrodite flowers in both varieties contained five anthers (Supplemental Figure S1N). During the ripening process, the color of the sepals, which enclosed the grain, changed from green to yellow in Longli-4, while for CA3-1, the color did not change until the grain was ready to harvest, and the grain had a dark red or purple color on its surface (Supplemental Figure S1O).

### 2.2. Statistics of Genomic Variances in Longli-4 and CA3-1 against the Reference Genome (PI614886)

**Figure 2:**
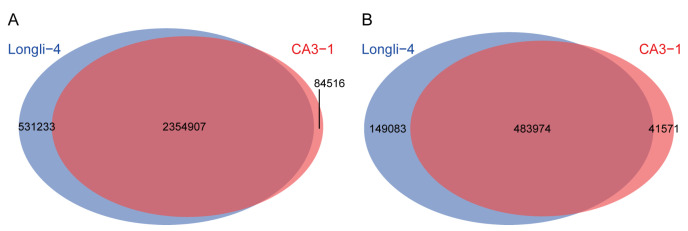
Total chromosome-targeted SNPs and INDELs. (**A**,**B**) The total SNPs (**A**) and INDELs (**B**) when both Longli-4 and CA3-1 were included for variation calling. Numbers in sky blue ellipses represent that SNPs and INDELs had no read-support information in Longli-4 and were assigned as ‘N’. Numbers in light coral ellipses represent variances with missing information in CA3-1. Numbers with color overlap show that these SNPs and INDELs were identified in both Longli-4 and CA3-1.

**Figure 3:**
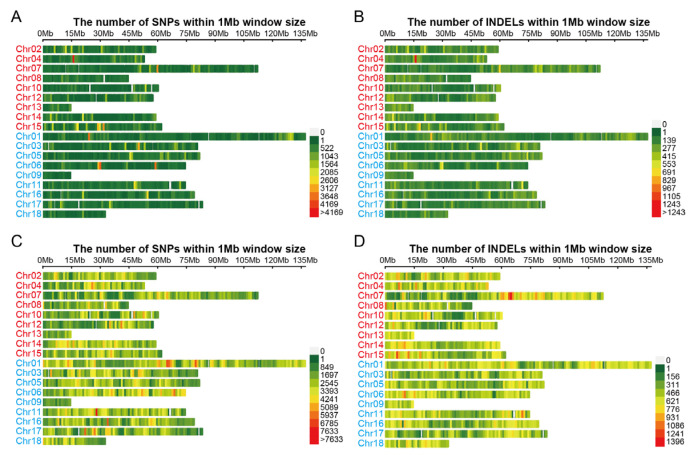
Chromosome distribution patterns of total variations identified individually in Longli-4 and CA3-1 against the reference genome (PI614886). (**A**,**B**) SNPs (**A**) and INDELs (**B**) of Longli-4 on each chromosome. (**C**,**D**) SNPs (**C**) and INDELs (**D**) of CA3-1 on each chromosome. The A sub-genome of quinoa includes Chr02, Chr04, Chr07, Chr08, Chr10, Chr12, Chr13, Chr14 and Chr15 and is shown in red, while the B sub-genome includes Chr01, Chr03, Chr05, Chr06, Chr09, Chr11, Chr16, Chr17 and Chr18 and is shown in blue.

A total of 3,149,050 single nucleotide polymorphisms (SNPs) and 711,178 small insertion and deletion (INDELs) were found when both Longli-4 and CA3-1 were included for variance calling. After filtering out the SNPs and INDELs that were not mounted to the chromosome, a total of 2,970,656 SNPs and 674,628 INDELs were obtained (Figure 2). To identify the genomic variation in each variety, the SNP and INDEL datasets were screened individually for Longli-4 and CA3-1. After filtering the variances with no read support (e.g., ‘N’) and with the same genotype compared with the reference line PI614886, a total of 407,651 SNPs and 212,724 INDELs were detected in Longli-4. The variation numbers of CA3-1 were greatly higher than those in Longli-4, and 2,731,411 SNPs and 587,935 INDELs were found, suggesting that Longli-4 is more closely related to PI614886. The distribution patterns of SNPs and INDELs were drawn on each chromosome based on the position information (Figure 3). The total SNP numbers from B sub-genome (Chr01, 03, 05, 06, 09, 11, 16, 17, and 18) were higher than those from A sub-genome (Chr02, 04, 07, 08, 10, 12, 13, 14, and 15) in both varieties (Figure 3A,C and Supplemental Table S1). The SNPs were not evenly distributed on each chromosome, and the hot regions of the SNP occurrence were totally different in both varieties (Figure 3A,C). For instance, Chr04 from the A sub-genome had the hottest region in Longli-4, whereas in CA3-1, it was Chr11 from the B sub-genome. Partly due to the shortest chromosome characteristics in the A and B sub-genomes, Chr13 and Chr09 were the chromosomes containing the least numbers but the highest densities of SNPs in both varieties (Supplemental Table S1). Similarly, the INDEL numbers from the B sub-genome were higher than those from the A sub-genome in both varieties, but the numbers of INDELs per 10 kb window on each chromosome were two and five in Longli-4 and CA3-1, respectively (Figure 3B,D and Supplemental Table S1). The INDEL hot region occurred on Chr04 in Longli-4 but on Chr07 in CA3-1 (Figure 3B,D).

**Figure 4:**
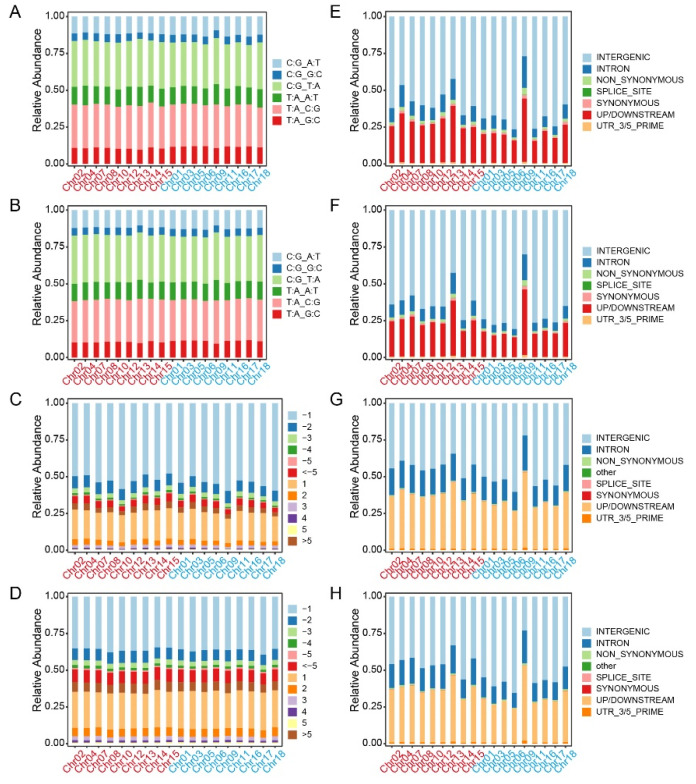
Stacked bar charts of total variations in each of Longli-4 and CA3-1. (**A**,**B**) SNP variations are categorized into six groups based on transitions and transversions, and the percentage of each group on each chromosome in Longli-4 (**A**) and CA3-1 (**B**) is presented as a stacked bar. (**C**,**D**) INDELs including insertions and deletions are classified into twelve groups based on reference genome information, and proportions of twelve groups on each chromosome in Longli-4 (**C**) and CA3-1 (**D**) are both stacked as 100%. (**E**–**H**) SNPs (**E**,**F**) and INDELs (**G**,**H**) are categorized into seven groups based on annotation, and relative abundance values of seven groups on each chromosome in Longli-4 (**E,G**) and CA3-1 (**F,H**) are shown as a stacked bar. The chromosomes from the A and B sub-genomes of quinoa are shown as those in Figure 3.

Generally, transitions are defined as the substitution between two purines (A/G) or two pyrimidines (C/T), whereas the interchanges of purines with pyrimidines (G/T, A/T, A/C, G/C) are assigned as transversions. The main SNPs were transitions, accounting for 59.27% and 59.77% of the total variations, which were obviously higher than the transversions in Longli-4 and CA3-1, respectively (Figure 4A,B and Supplemental Figure S2A,B). For the transitions, the proportion of C/T SNPs was higher than that of A/G in both varieties. For the transversions, the relative abundance values of G/T, A/T, and A/C SNPs were similar, whereas the G/C SNPs accounted for the lowest proportion of the total SNPs in both varieties (Figure 4A,B and Supplemental Figure S2A,B). In comparison with the reference genome, the INDELs can be classified into 12 categories based on length variation (Figure 4C,D and Supplemental Figure S2C,D). Deletions accounted for a higher proportion than that of insertions (Figure 4C,D and Supplemental Figure S2C,D). Approximately 53% of the total INDELs in Longli-4 were identified as mononucleotide deletion, whereas the mononucleotide insertion only accounted for 19.24%. In CA3-1, the mononucleotide deletion and insertion made up 36.09% and 25.01%, respectively. According to the snpEFF annotations, most SNPs and INDELs occurred in the intergenic regions (64.54% vs. 70.36%; 48.05% vs. 51.78%), while 23.04% and 14.59% SNPs and 33.80% and 32.02% INDELs located in upstream/downstream regions in Longli-4 and CA3-1, respectively. Detailed analyses revealed that only 1.76% and 1.35% SNPs and 0.57% and 0.75% INDELs resulted in non-synonymous mutations in Longli-4 and CA3-1, respectively (Figure 4E–H and Supplemental Figure S2E–H).

### 2.3. Genetic Divergence between Longli-4 and CA3-1

**Figure 5:**
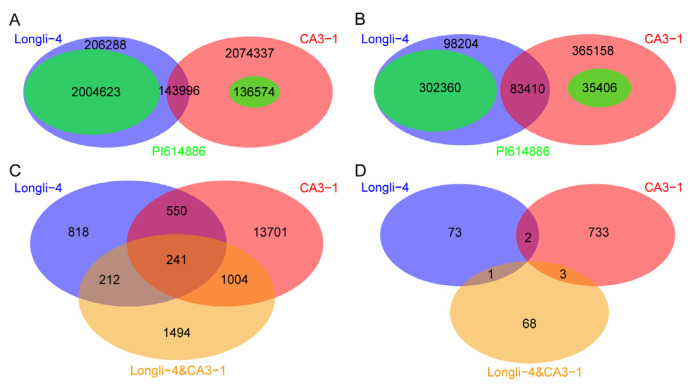
Genetic variations between Longli-4 and CA3-1. (**A**,**B**) Numbers of SNPs (**A**) and INDELs (**B**) between Longli-4 and CA3-1. The variety-specific SNPs and INDELs for Longli-4 and CA3-1 are shown in blue and red, respectively. Blue + red represents the shared SNPs (143,996) or INDELs (83,410) between Longli-4 and CA3-1. Green indicates that the SNP or INDEL variations have the same genotype between one variety and the reference line PI614886. (**C**,**D**) Genes with pairwise non-synonymous SNPs and INDELs variation (**C**) and stop-gain SNPs variation (**D**) between Longli-4 and CA3-1. Blue (818; 73) and red (13701; 733) indicate that the gene set with non-synonymous/stop-gain variations is specifical from Longli-4 and CA3-1, respectively. Yellow (1494; 68) represents that the non-synonymous/stop-gain variations are shared between Longli-4 and CA3-1. Blue + red (550; 2) represents that Longli-4 and CA3-1 variations result in the mutation of the same gene, but in different positions. Blue + yellow (212; 1) represents that the gene variation is caused by Longli-4 alone or by different variants of Longli-4 and CA3-1 at the same position. Red + yellow (1004; 3) represents that the gene variation is caused by CA3-1 alone or by different variants of Longli-4 and CA3-1 at the same position. Blue + red + yellow (241) contains all variation types.

**Figure 6:**
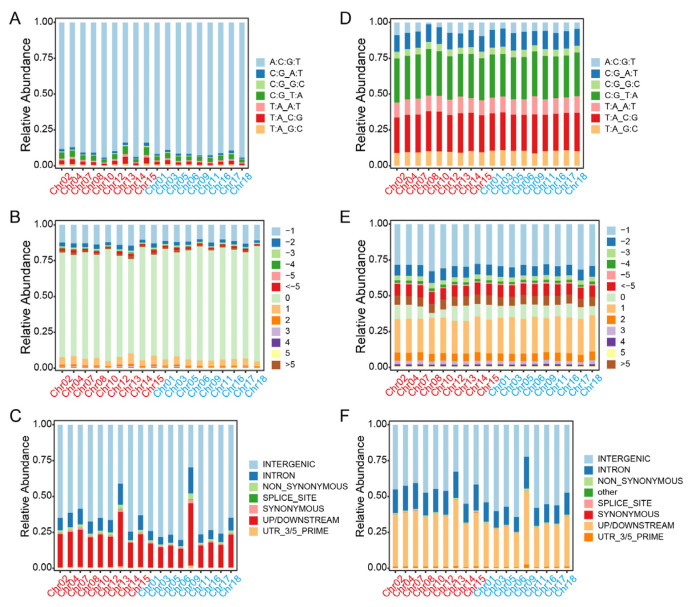
Stacked bar charts of the pairwise SNPs and INDELs in Longli-4 and CA3-1. (**A**,**D**) Percentages of the seven SNP groups on each chromosome in Longli-4 (**A**) and CA3-1 (**D**). The group of ‘A:C:G:T’ in (**A**) shows the relative abundance of SNPs (2,004,623) with the same nucleotide bases in Longli-4 and the reference line as that in Figure 5A, and the other six groups are classified based on transitions and transversions. The same category method is also used in (**D**) for CA3-1. (**B**,**E**) Percentages of the 13 INDEL groups on each chromosome in Longli-4 (**B**) and CA3-1 (**E**). The group of ‘0’ in (**B**) represents the relative abundance of INDELs (302,360) with the same genotypes in Longli-4 and the reference line as that in Figure 5B, and the other twelve groups are classified based on the length of insertions and deletions. The same groups are also used in (**E**) for CA3-1. (**C**,**F**) SNPs (**C**) and INDELs (**F**) are classified into seven groups based on annotation information, and relative abundance values of all groups on each chromosome are shown as a stacked bar. The chromosomes from the A and B sub-genomes of quinoa are shown as those in Figure 3.

**Figure 7:**
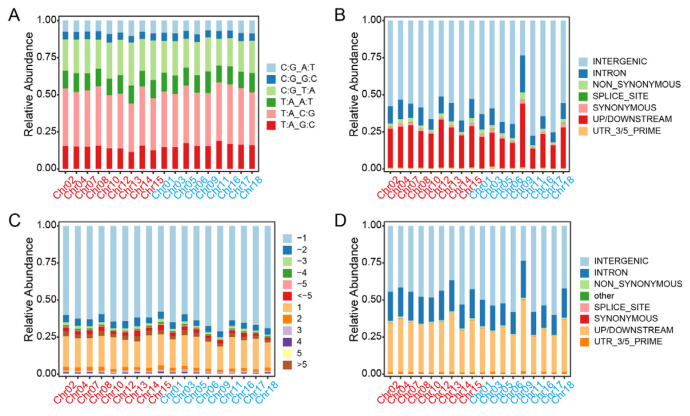
The shared SNPs and INDELs in Longli-4 and CA3-1. (**A**) SNP variations including transitions and transversions are categorized into six groups, and the percentage of each group on each chromosome is shown as a stacked bar. (**B**,**D**) SNPs (**B**) and INDELs (**D**) are classified into seven groups based on annotation, and percentages of the seven groups on each chromosome are shown as a stacked bar. (**C**) INDELs including insertions and deletions are classified into twelve groups according to the reference genome information, and percentages of the twelve groups on each chromosome are stacked as 100%. The chromosomes from the A and B sub-genomes of quinoa are shown as those in Figure 3.

Among the total obtained 2,970,656 SNPs, 2,354,907 SNPs were detected in both Longli-4 and CA3-1, whereas the SNPs with missing information in Longli-4 and CA3-1 were 531,233 and 84,516, respectively (Figure 2A and Figure 5A). Similarly, 483,974 out of 674,628 INDELs were identified in both varieties, and the numbers with no read support in Longli-4 and CA3-1 were 149,083 and 41,571, respectively (Figure 2B and Figure 5B). The SNPs and INDELs can be further classified into different categories when the genomic information of each variation from the reference line was included. For instance, there were 143,996 SNPs having the same nucleotide bases in both varieties but different against the reference bases at the same positions, suggesting that these SNPs are shared in Longli-4 and CA3-1 (hereafter named ‘shared SNPs’, Figure 5A). For the rest of the SNPs (hereafter named ‘pairwise SNPs’), approximately 91% of SNPs (2,004,623) had the same nucleotide bases in Longli-4 and PI614886 but with different bases in CA3-1. In contrast, this kind of SNPs only accounted for 6.17% (136,574) in CA3-1. These results further imply that CA3-1 is in a distant relationship with both Longli-4 and the reference line. Additionally, there were 206,288 SNPs in Longli-4 and 2,074,337 SNPs in CA3-1 having different nucleotide bases in both varieties, and in these variety-specific SNPs, transitions (A/G and C/T) accounted for approximately 60% in Longli-4 and CA3-1 (Figure 5A and Figure 6A,D and Supplemental Figure S3A,D). The same procedure was conducted for INDEL classification and similar results were generated (Figure 5B). In Longli-4, 75.32% of INDELs (302,360) had the same genotypes with the reference, but only 8.59% in CA3-1. Among the variety-specific INDELs (98,204 vs. 365,158), mononucleotide insertion and deletion were the predominant types. Mono- and di-nucleotide deletions were two to three times as greater as that of insertions in Longli-4. In CA3-1, mono- and di-nucleotide deletions were also higher than that of insertions, but to a lesser extent (Figure 6B,E and Supplemental Figure S3B,E). After excluding the shared variations (143,996 SNPs and 83,410 INDELs), pairwise SNPs and INDELs mainly occurred in the intergenic regions, which can reach up to 70.64% and 50.99%, respectively. The following was in INTRON regions, accounting for 7.37% and 14.55%, and the non-synonymous variations only made up 1.43% and 0.73%, respectively (Figure 6C,F and Supplemental Figure S3C,F). Likewise, the shared SNPs (143,996) and INDELs (83,410) between Longli-4 and CA3-1 had similar distribution patterns respect to the variation categories and annotation effects (Figure 7 and Supplemental Figure S4).

**Figure 8:**
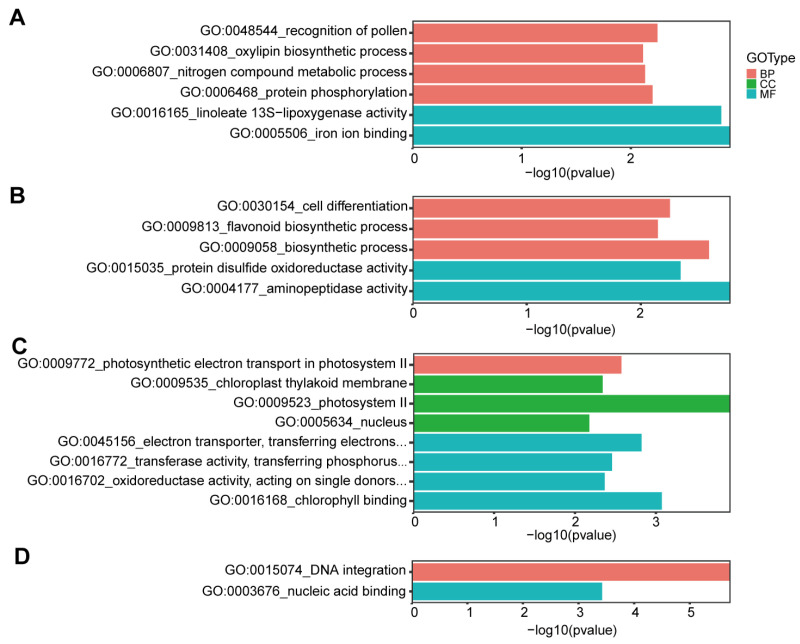
GO enrichment of the genes with the non-synonymous and stop-gain variations in Longli-4 and CA3-1. (**A**–**C**) GO terms of the non-synonymous genes specific in Longli-4 (**A**), in CA3-1 (**B**), and shared between Longli-4 and CA3-1(**C**). (**D**) GO terms of the stop-gain genes specific in CA3-1.

A total of 40,082 genes contained pairwise or shared SNPs/INDELs, among which 11,750 had synonymous variations, 18,020 had non-synonymous variations (Figure 5C). There were 880 genes which had a premature stop codon in SNPs variations (Figure 5D). The genes including non-synonymous variations can be divided into three categories: (1) 818 genes with the Longli-4 specific variations; (2) 13,701 genes with the CA3-1 specific variations; (3) 3501 genes containing Longli-4 and CA3-1 variations at same and/or different positions. GO enrichment analyses were then performed for these genes. For category one, GO terms ‘recognition of pollen’ (GO:0048544, *p* = 0.0057), ‘oxylipin biosynthetic process’ (GO:0031408, *p* = 0.0077), ‘nitrogen compound metabolic process’ (GO:0006807, *p* = 0.0074), ‘protein phosphorylation’ (GO:0006468, *p* = 0.0063), ‘linoleate 13S-lipoxygenase activity’ (GO:0016165, *p* = 0.0015), and ‘iron ion binding’ (GO:0005506, *p* = 0.0012) were specifically enriched (Figure 8A). For type two, five GO terms, including ‘cell differentiation’ (GO:0030154, *p* = 0.0056), ‘flavonoid biosynthetic process’ (GO:0009813, *p* = 0.0071), ‘biosynthetic process’ (GO:0009058, *p* = 0.0025), ‘protein disulfide oxidoreductase activity’ (GO:0015035, *p* = 0.0045), and ‘aminopeptidase activity’ (GO:0004177, *p* = 0.0016), were enriched (Figure 8B). Specifically, GO terms ‘photosynthetic electron transport in photosystem II’ (GO:0009772, *p* = 0.0027), ‘photosystem II’ (GO:0009523, *p* = 0.0001), ‘chloroplast thylakoid membrane’ (GO:0009535, *p* = 0.0045), ‘nucleus’ (GO:0005634, *p* = 0.0066), ‘chlorophyll binding’ (GO:0016168, *p* = 0.0008), ‘electron transporter, transferring electrons within the cyclic electron transport pathway of photosynthesis activity’ (GO:0045156, *p* = 0.0015), ‘transferase activity, transferring phosphorus- containing groups’ (GO:0016772, *p* = 0.0035), and ‘oxidoreductase activity, acting on single donors with incorporation of molecular oxygen, incorporation of two atoms of oxygen’ (GO:0016702, *p* = 0.0043), were enriched for the third type of genes (Figure 8C). Meanwhile, GO enrichment analyses were carried out for the genes with stop-gain variations in three categories. Only two enriched GO term including ‘DNA integration’ (GO:0015074, *p* = 1.85 × 10^−6^) and ‘nucleic acid binding’ (GO:0003676, *p* = 0.0004) were found in category two (Figure 8D).

### 2.4. Variation of Candidate Genes Relevant to Vital Agronomic Traits and Domestication

To explore the genetic changes underlying some important agronomic traits in Longli-4 and CA3-1 during breeding, 7 MEP (2-C-methyl-_D_-erythritol 4-phosphate) pathway-, 9 flavonoid-, 72 betalain-, 352 flowering-, 226 seed size-, 151 domestication-, and 34 saponin biosynthesis-related genes from the model plant *Arabidopsis thaliana* and various staple crops as well as other plants, were used as seeds to identify the homologous genes in quinoa. As a result, out of the 18,020 non-synonymous genes, only 414 homologous genes were obtained by homologous sequence alignment. Among which, 1 betalain gene, 7 genes for flowering, 3 genes for seed size, and 1 gene for domestication had the Longli-4-specific non-synonymous variations, while 6 MEP-pathway genes, 6 flavonoid genes, 8 betalain genes, 153 flowering genes, 73 seed size genes, 47 domestication genes, and 13 saponin genes had the CA3-1-specific non-synonymous mutations. Furthermore, 1 MEP-pathway gene, 2 flavonoid genes, 2 betalain genes, 40 flowering genes, 20 seed size genes, 13 domestication genes, and 3 saponin genes having non-synonymous variations, were shared by Longli-4 and CA3-1, and only one seed size gene homologous of *CKX1* (*cytokinin oxidase/dehydrogenase 1*) was found with stop-gain mutations in both varieties (Table 1 and Supplemental Table S2).

### 2.5. Heterozygosity Rates of Two Varieties

**Figure 9:**
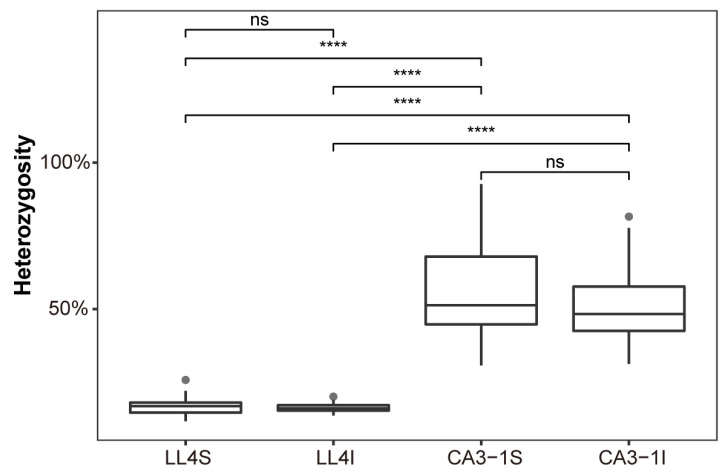
The heterozygosity of SNP and INDEL. LL4S represents the SNPs of Longli-4. LL4I represents the INDELs of Longli-4. CA3-1S represents the SNPs of CA3-1. CA3-1I represents the INDELs of CA3-1. ns, not significant, ****, *p* < 10^−6^.

The homogeneity during the individual selection process is dependent on natural mutation, hybridization, and cultivation methods. In this study, Longli-4-3 and CA3-1-3 were individually harvested from the Haiyuan plots [36], and the descendants were selected for genome sequencing. Based on the SNPs and INDELs detected separately in two varieties (Figure 3), heterozygosity rates were measured according to the whole chromosomes. The whole average heterozygosity of Longli-4 calculated based on SNPs was 16.73%, ranging from 11.63% to 25.76%, while for INDELs, it was 16.34%, ranging from 13.58% to 20.06%. However, in CA3-1, the heterozygosity rates were 57.84% (30.70–92.72%) and 52.01% (31.23–81.58%) according to SNPs and INDELs (Figure 9). Similar results were also obtained from the comparisons within each sub-genome (Supplemental Figure S5). These results indicate that the Longli-4 has achieved higher homogeneity values than CA3-1.

Since only one seedling for each variety was used for sequencing, the heterozygosity rates could be overestimated. Individual plants harvested from different places with different cultivation methods were therefore used to determine the heterozygosity rates. Individual heterozygosity was calculated by the frequency of heterozygous loci within 48 offsprings using INDEL markers from nine chromosomes (Table 2 and Supplemental Figure S6). Longli-4-1, Longli-4-2, CA3-1-1 and CA3-1-2 were from Tianzhu county, and the heterozygosity values were 0–4.26%, 0%, 0%, and 0–2.08%, respectively. On the contrary, individuals from Haiyuan county showed higher heterozygosity rates, and the highest values of Longli-4-3 and CA3-1-3 can reach up to 28.26% and 25%, respectively. The same markers were also used to test the heterozygosity of the commercial grains of Longli-4-4. Results with 98 seedlings of Longli-4-4 revealed that the heterozygosity rates ranged from 0 to 5.21%. These results suggest that cultivation methods strongly affect the homogeneity of each variety, and adequate spaces are necessary to maintain the heterozygosity at a lower level. However, three genotypes (i.e., AA, AB, and BB) were detected with the markers of Chr15, Chr16, and Chr17 in Longli-4-3 (Supplemental Figure S6), and the genetic effect in parental lines could also contribute to the high heterozygosity rates of Longli-4 variety.

## 3. Discussion

Analyzing the genomic variation pattern of quinoa is very important to understand its phenotypic variation and will provide guidance for quinoa breeding. In this study, higher numbers of SNPs and INDELs were detected in CA3-1, implying that CA3-1 is more distantly related to the reference line compared with the Longli-4. While the numbers and the distribution patterns of SNPs varied significantly in two varieties, the main variation patterns of the SNPs were the same (C/T or G/A), suggesting that the transition variation is the most frequent base substitution during species evolution and variety development. The same phenomenon was also discovered in other species, such as *Arabidopsis* [37] and rice [38].

Considering various factors including environmental adaptability and breeding goals, germplasm resources may maintain different genetic variation under different selection pressures [39]. Accordingly, the shared SNPs between Longli-4 and CA3-1 only account for 6%, which is far less than the 94% pairwise SNPs (Figure 5A). A total of 13,701 non-synonymous genes were attributed to the CA3-1 specific mutations and the enriched GO term ‘flavonoid biosynthetic process’ (GO:0009813) may provide some evidence for the difference in seed and stem color between Longli-4 and CA3-1 [31]. Moreover, crop yield is largely determined by the efficiency of photosynthesis [40,41]. For the 3501 shared non-synonymous genes, the related elements in the process of photosynthesis were significantly enriched (Figure 8C). This result is largely correlated with the relatively high yields of Longli-4 and CA3-1 [31], and is consistent with the need for high yield in the breeding programs [40,42,43].

To further explore the genetic differences between CA3-1 and Longli-4 in the breeding process, sets of candidate genes related to some important agronomic traits were selected. Among them, 13 genes with the variety-specific stop-gain variation may contribute to the phenotypic differences between Longli-4 and CA3-1 (Supplemental Table S2). For instance, only one domestication gene (AUR62007281) is mutated to obtain a premature stop codon in Longli-4, and its sequence identity against the *Ramosa1* (*Ra1*) gene is 33.6%. Loss of function mutation in maize *Ra1* gene results in extra and longer inflorescence branches and heterologous expression of the *Ra1* gene in *Arabidopsis* increases the size of reproductive organs [44,45,46]. However, for the specific stop-gain variations of the CA3-1,the AUR62035617 gene with 68.6% sequence identity value against *Waxy 1* (*WX1*) was identified. *WX1* is an important agronomic trait-related gene playing a vital role in grain quality, especially for the amylose content of the grains [47]. In the rice, *wx1* mutant produces the opaque, semitranslucent, or transparent grains [47].

Seed size is a crucial agronomic trait for breeding and directly affects grain yield [36]. AUR62037832 and AUR62003488, with 57.6% and 57.4% sequence identity values against the *TTG2* and *GS9*, respectively, were identified for the CA3-1-specific stop-gain variation. The TTG2 (Transparent Testa Glabra2), a WRKY family transcription factor, can promote cell expansion in maternal tissues and thereby increase the seed size [48]. GS9 (Grain Shape Gene on Chromosome 9) plays an important role in seed formation, mainly controlling the seed shape, and the *gs9* null mutant produces the slender grains [49]. The divergence in these two genes may underly the yield difference between Longli-4 and CA3-1 [31]. Interestingly, AUR62026114 was found to contain the stop-gain variants in both varieties. This gene is the homolog of the *CKX1* gene, which negatively regulate the number of grains by degrading the cytokinins [50]. Therefore, *CKX1* should be the common target selected in both Longli-4 and CA3-1 breeding processes to increase the yield values.

Generally, plant pigments, flavonoids/anthocyanins, betalains, and carotenoids, are essential for the colorful appearance of plant organs [51,52]. Previous studies have revealed that the WRKY44 transcription factors can regulate the expression of the *cytochrome P450-like1* and thereby control the betalain biosynthesis [53,54]. AUR62037832, a homolog of *WRKY44*, had a premature stop codon in CA3-1. The mutation of AUR62037832 may result in the different colors of the seed and stem between Longli-4 and CA3-1.

Flowering is an essential agronomic trait to be considered during the breeding process. The Unusual floral organs (UFO) is important for maintaining the normal development of floral [55]. The cytochrome P450 monooxygenases CDP/CYP90A1, is a key rate-limiting enzyme for the biosynthesis of brassinosteroids [56,57], which is involved in pollen tube development and promotion of flowering [58,59]. Delay of germination 1 (DOG1) acts in coordination with the abscisic acid (ABA) to delay seed germination [60] and is also essential for the flowering time regulation [61]. The AUR62036157, AUR62004597, and AUR62006898 are homologous genes of *UFO*, *CDP/CYP90A1*, and *DOG1* in quinoa with the sequence identity values of 62.3%, 60.3%, and 28.9%. The stop-gain variants found in these three genes might affect the inflorescence morphology and flowering time of CA3-1, but the real effects remain to be confirmed.

High saponin content in the outer layer of quinoa grains makes it a major limiting factor for quinoa as a table food [6,62]. Four saponin-related genes, AUR62025671, AUR62031838, AUR62025695, and AUR62025696, were identified to contain the CA3-1-specific stop-gain variations. These four gene were predicted to encode the beta-amyrin synthase (BAS) proteins, which are the key enzymes in saponin synthesis pathway [19]. These genes’ mutations may influence the saponin accumulation, and will be the key candidates for further investigation of saponin components in CA3-1 grains.

In this study, there were many other variances possibly leading to the genetic divergence between Longli-4 and CA3-1 (Supplemental Table S2). These candidate genes may have important reference significance for further quinoa breeding. Since only a single individual from each of the varieties was sequenced, the number of candidate genes may be overestimated due to the difference in genetic background itself. More individual plants from different places with different cultivation methods are required to validate the candidate genes in future experiment.

In conclusion, conventional breeding has been widely used to develop quinoa cultivars [25,26,27,28,29,30,31,32]. However, mass selection, individual selection, and hybridization are highly time costed and can only select the plants with obvious phenotypic divergence. Marker-assisted breeding can greatly accelerate the breeding process through genotype screening at very early growth stage. The genetic variations between Longli-4 and CA3-1 presented in this study may provide guidance for the molecular-assisted breeding of quinoa in China.

## 4. Materials and Methods

### 4.1. Plant Materials

To measure the heterozygosity rates of Longli-4 and CA3-1, individual plants from different sites with different cultivation methods were selected. For example, a total of 150 tested varieties including Longli-4 and CA3-1 were sown in Tianzhu county, Gansu province, by the Gansu Academy of Agricultural Sciences in 2019. Each variety had four separate rows with a distance of 40 cm, and each row was approximately 20 m. For each variety, five plants with similar phenotypes were harvested individually, and two of them (e.g., Longli-4-1 and -2 for Longli-4, and CA3-1-1 and -2 for CA3-1) were randomly selected for the following experiments. Longli-4-3 and CA3-1-3 came from the Agricultural Technology Extension and Service Center of Haiyuan County. Plot tests with Longli-4, CA3-1 and the other five varieties were conducted in the Haiyuan dryland in 2019 [31]. Each plot was 2.25 m^2^ (1.5 m × 1.5 m) with five rows, and the distance between the two plots was 50 cm. For each variety, four replicated plots were used to evaluate the agronomic performance, and one single plant (e.g., Longli-4-3 for Longli-4, and CA3-1-3 for CA3-1) was randomly sampled for the tests in this study. Approximately 50 seeds from each of the selected plants were germinated on soaked filter papers, and the genomic DNA was extracted from each of the 48 seedlings individually. Since Longli-4 has been a commercial variety and the identity of seed multiplication is supposed to be homogeneity. The commercial seeds of Longli-4 were also included to test the heterozygosity rate, and 96 seedlings named Longli-4-4 were sampled for DNA extraction.

### 4.2. Phenotype Comparison

Seeds of Longli-4-2 and CA3-1-1 were sown in nutrient soil (Pindstrup, Denmark) in a PVC pot with a diameter of 5 cm and a depth of 10 cm, and water was supplied twice a week. The growth condition of the chamber was 14 h light/10 h dark. Four-month-old plants were used to measure the plant height, internode length, lateral branch number, stem diameter, leaf area, main panicle length, and main panicle diameter. The data were drawn in boxplots using the *ggplot2* R package [63].

### 4.3. Genome Resequencing

Seeds of Longli-4-3 and CA3-1-3 were sowed into the pots filled with a mixture of nutrient-soil and loess (1:1), 5 cm in diameter, 10 cm in height, and then transferred into a common garden. Young leaves from individual plants in the common garden were sampled and stored in liquid nitrogen for DNA extraction. The sequencing libraries were constructed according to the standard Illumina protocol, and the paired-end sequencing was performed using the Illumina platform at Biomarker Technologies (Beijing, China). A total of 74,878,963 and 80,381,530 clean reads were obtained with Q30 values of 93.84% and 94.56% for Longli-4 and CA3-1, respectively. The clean reads were aligned back to the quinoa reference genome (PI614886) [19] for subsequent variation analyses. The properly mapped ratios were 97.47% and 96.12% for Longli-4 and CA3-1, respectively.

### 4.4. SNP and INDEL Calling and Statistical Analyses

After filtering the redundant reads by Picard (http://sourceforge.net/projects/picard/; version 1.94 (accessed on 24 August 2020)), SNP and INDEL calling were performed using GATK (version 3.8) with default setting [64]. Variations in Longli-4 and CA3-1, which cannot be mounted into the 18 pseudochromosomes, were filtered out. SNPs and INDELs in Longli-4 and CA3-1 against PI614886 were independently analyzed using RStudio, and the distribution patterns of SNPs or INDELs on each chromosome were presented by the *CMplot* R package [65]. Statistics of the number and the relative abundance of SNPs and INDELs were measured based on variation types and genomic positions using *do* [66] and *data.table* [67] R packages, and the *ggplot2* R package [63] was employed to draw the stacked bar charts.

To identify the genetic variation between Longli-4 and CA3-1, the whole SNPs and INDELs were categorized into three groups based on the position information of the reference genome. The SNPs and INDELs classified into Longli-4- and CA3-1-specific groups were excluded from the following analyses since the information on these variances was lacking in one of the tested varieties. SNPs and INDELs which were at the same genome positions and detected in both varieties, were retained for the variation comparison between Longli-4 and CA3-1. The numbers of SNPs and INDELs showed the same and different variations between Longli-4 and CA3-1 were counted using *do* [66] and *data.table* [67] R packages, and results were shown in *venn.plots* by *ggvenn* R package. Among them, the variation numbers with the same genotypes against the reference genome were highlighted for each variety in SNP and INDEL *venn.plots*.

### 4.5. GO Enrichment Analyses and Variation in Candidate Genes

The SNP/INDEL effect was annotated using SnpEff [68] according to the reference genome annotation. For each variety, the genes with non-synonymous variation and stop-gain mutations were identified, and GO enrichment analyses with a threshold of 0.01 were then performed using *clusterProfiler* package of R [69]. Results were shown in histograms using the *ggplot2* package of R [63].

MEP, flavonoid, and flowering genes in *Arabidopsis thaliana* [70,71,72], betalain genes in *Beta vulgaris*, *Mirabilis jalapa*, and quinoa [54,73,74,75,76,77], seed size genes in *Arabidopsis* and crop species [36], domestication genes in diverse crops [78,79,80,81,82], and saponin genes reported in quinoa [19] were used as the seeds to identify the homologous genes in quinoa genome by an all-against-all blast approach [83]. Only the genes with a reciprocal best hit were retained for variation analyses. Based on the SNP/INDEL annotation data, genes with synonymous and non-synonymous mutations, and stop-gain variations in Longli-4 and CA3-1 were characterized, respectively. Statistics of variations of the candidate genes in Longli-4 and CA3-1 were summarized using the *do* and *data.table* packages of R [66,67].

### 4.6. Heterozygosity

Homozygous or heterozygous SNPs and INDELs were identified using RStudio. According to the filtered SNP and INDEL results, the heterozygosity rates of Longli-4 and CA3-1 were calculated independently, and the boxplots of results were generated by the *ggplot2* package of R.

One-week-old seedlings were used for the evaluation of the heterozygosity rates in Longli-4 and CA3-1 lines, and DNA was extracted with a simple method. Briefly, a single seedling was put into a 2 mL microcentrifuge tube with two beads. After being frozen in liquid nitrogen, the material was ground into a fine powder using a Retsch Mixer Mill MM 400 (Germany). The powder was then suspended with 150 μL Tris-EDTA buffer solution and incubated at 90 °C for 10 min. The tube was subsequently centrifuged at 10,000 r/min for 2 min, and the supernatant was pipetted as the template for the following PCR assays. Forty-eight seedlings were sampled for Longli-4-1, -2, -3 and CA3-1-1, -2, -3, while for the commercial line Longli-4-4, ninety-six seedlings were used. Nine INDEL markers were developed to test the heterozygosity rate of each line and the genotype of each seedling was identified based on the length and number of PCR bands.

## Figures and Tables

**Table 1:** Genomic variations of candidate genes in Longli-4 and CA3-1.

	L_non	C_non	L&C_non	L_STOP	C_STOP	L&C_STOP
MEP	0	6	1	0	0	0
Flavonoid	0	6	2	0	0	0
Betalain	1	8	2	0	1	0
Flowering	7	153	40	0	4	0
Seed size	3	73	20	0	2	1
Domestication	1	47	13	1	1	0
Saponin	0	14	3	0	4	0

L_non: the Longli-4-specific non-synonymous genes. C_non: the CA3-1-specific non-synonymous genes. L&C_non: the shared non-synonymous genes between Longli-4 and CA3-1. L_STOP: the Longli-4-specific stop-gain genes. C_STOP: the CA3-1-specific stop-gain genes. L&C_STOP: the shared stop-gain genes between Longli-4 and CA3-1.

**Table 2:** The outcrossing rate (%) of Longli-4 and CA3-1 varieties.

	Chr01	Chr02	Chr03	Chr04	Chr08	Chr14	Chr15	Chr16	Chr17
Longli-4-1	0	2.08	4.26	0	0	0	4.26	4.26	4.26
Longli-4-2	0	0	0	0	0	0	0	0	0
Longli-4-3	6.25	13.04	12.77	28.26	2.08	6.38	NA	NA	NA
Longli-4-4	1.04	1.05	0	1.06	2.10	0	2.08	5.21	0
CA3-1-1	0	0	0	0	0	0	0	0	0
CA3-1-2	0	2.08	0	2.08	0	0	0	0	0
CA3-1-3	2.08	2.08	2.08	0	0	2.08	20.83	25	0

## Data Availability

The data presented in this study are openly available in NCBI with the BioSample number of SAMN31693738 for Longli-4 and SAMN31693739 for CA3-1.

## Supplementary Materials

The following supporting information can be downloaded at: https://www.mdpi.com/article/10.3390/ijms232214030/s1, Figure S1: Phenotype comparisons of two quinoa varieties; Figure S2: Stacked bar charts of variation numbers in each of Longli-4 and CA3-1; Figure S3: Stacked bar charts of numbers of the pairwise SNPs and INDELs in Longli-4 and CA3-1; Figure S4: The shared SNPs and INDELs in Longli-4 and CA3-1; Figure S5: Boxplots of the heterozygosity values of SNP and INDEL in two sub-genomes; Figure S6: Genotyping using nine INDEL markers; Table S1: The distribution of SNPs or INDELs; Table S2: The non-synonymous and stop-gain genes caused by the variations in Longli-4, CA3-1 or both of them.
