# Supplementary material for: Genomic Variation Underlying the Breeding Selection of Quinoa Varieties Longli-4 and CA3-1 in China
Source: Int J Mol Sci. 2022 Nov 14;23(22):14030. doi: 10.3390/ijms232214030 (PMC9693436; doi:10.3390/ijms232214030)

Chr01	Forward_primer	TTTAATGAACAAACACAATAGCAAG
	Reverse_primer	GCCTCAAAATACGAAATTTACACC

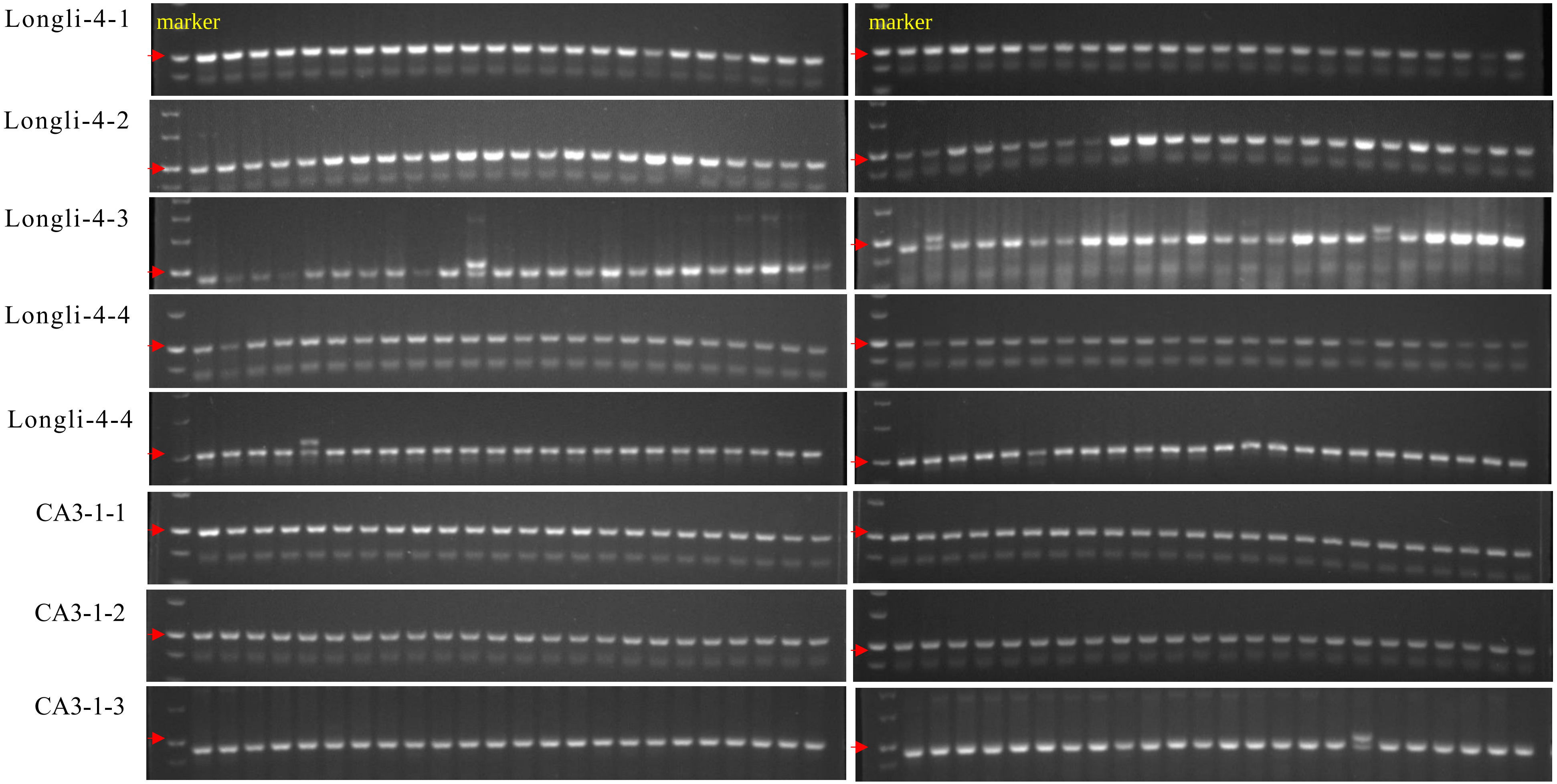

*The red arrow points to the position of 200 bp band, the same as below.

Chr02	Forward_primer	TTGCTCGAGACCAAGGTTTT
	Reverse_primer	TCTTCCACTGCAATCTGACG

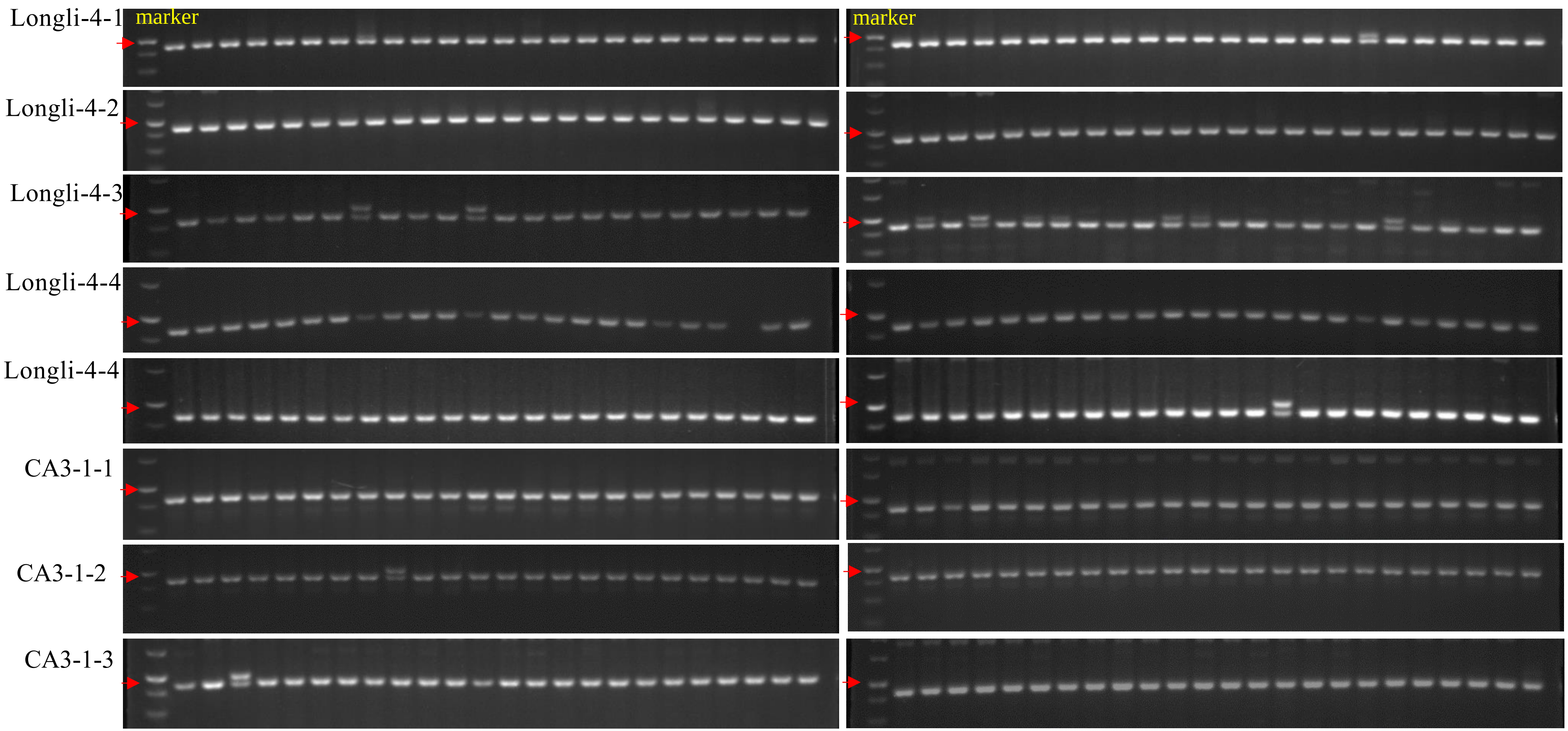

Chr03	Forward_primer	GAGGATTCCTGCCAATTTGA
	Reverse_primer	GCTTAAGGGGTTTGGATGGT

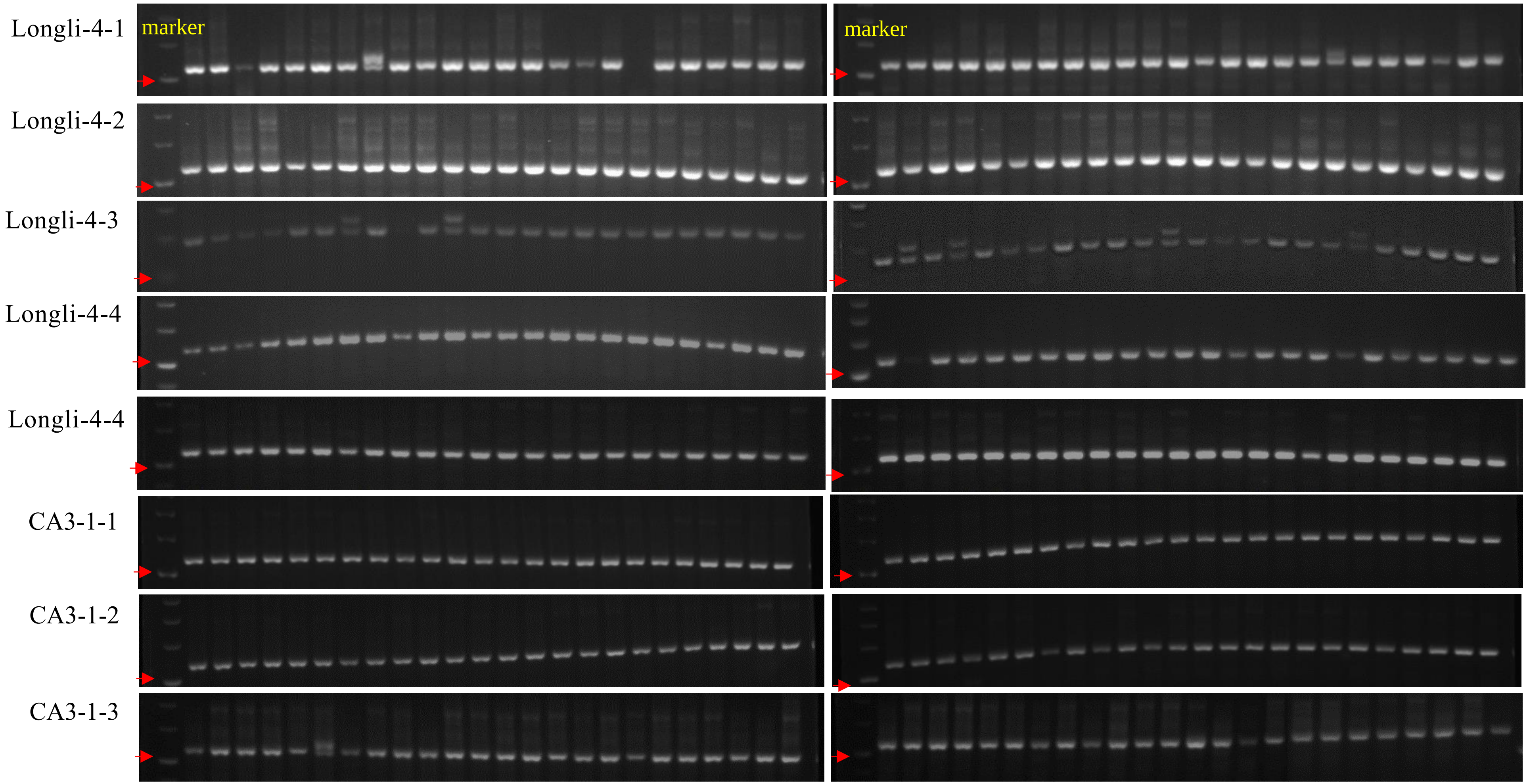

Chr04	Forward_primer	AACTGGGGCTAGCTCACTCA
	Reverse_primer	TTCCTCCACCTGTTTCGACT

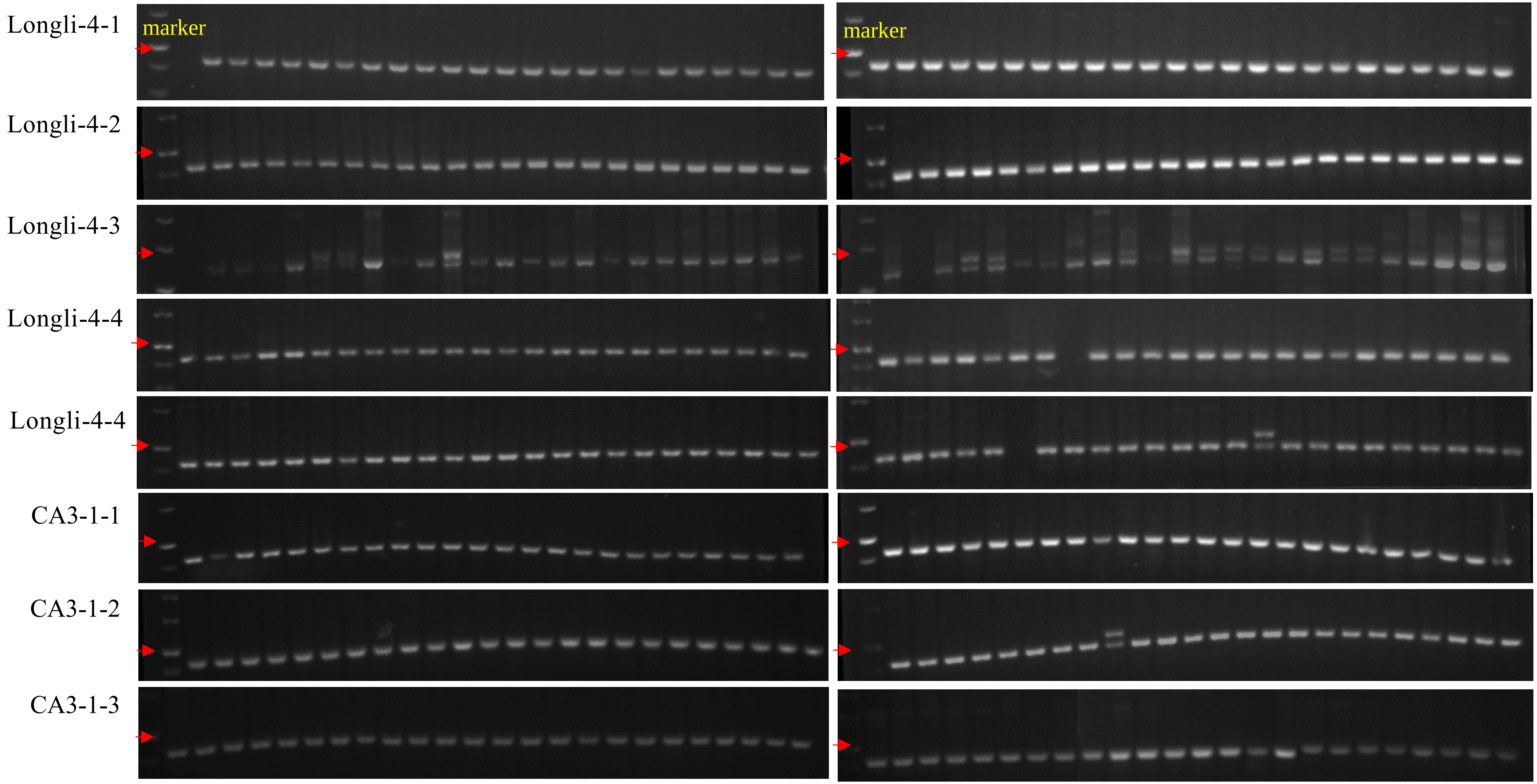

Chr08	Forward_primer	GGGTGGTGTTTGATGCTTCT
	Reverse_primer	ACCTTACCGCAGCACAGATT

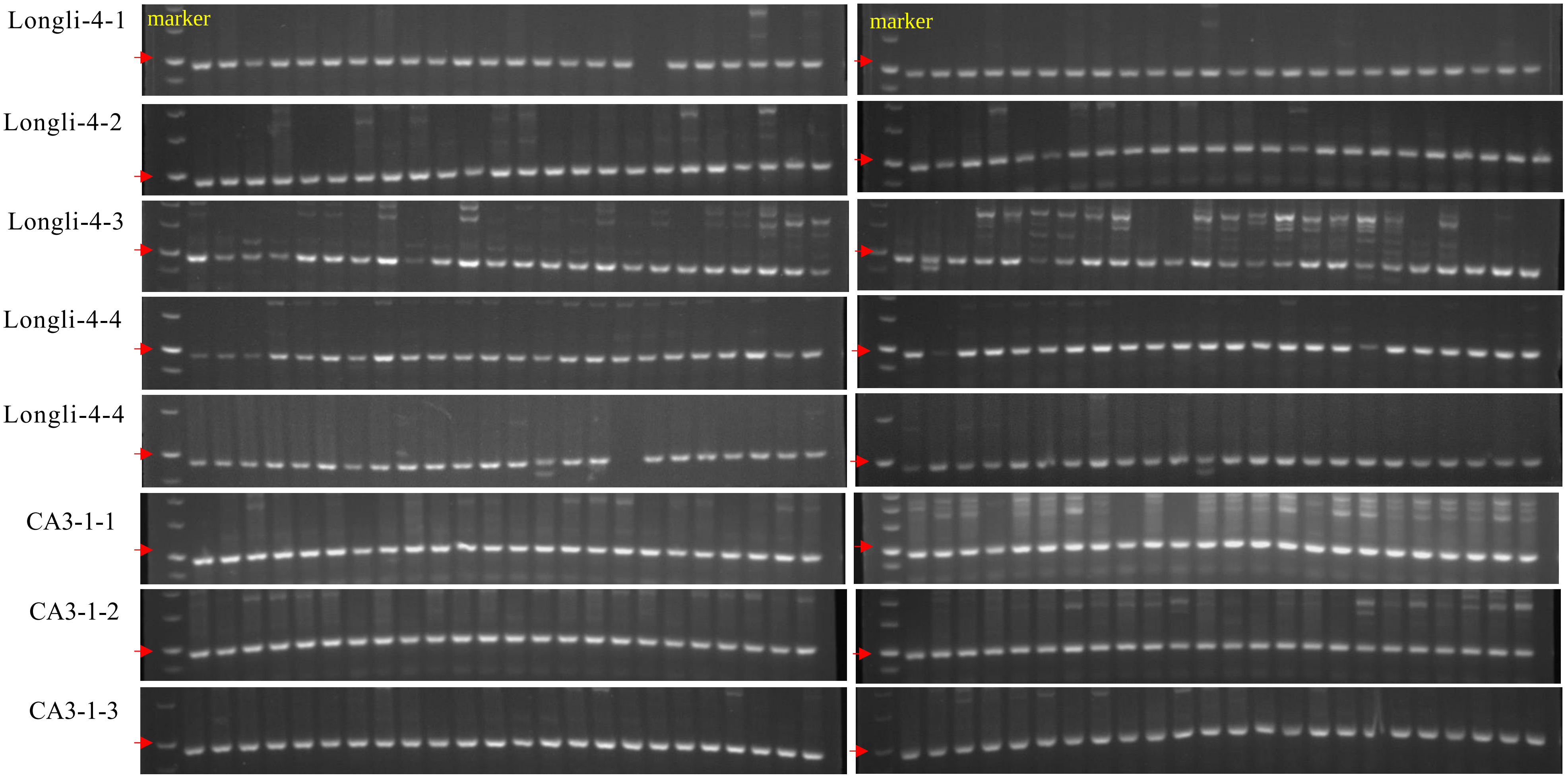

Chr14	Forward_primer	TCCACTTGTTTCCTCCTCCTT
	Reverse_primer	CGGATATGAAGTAACGATTACCA

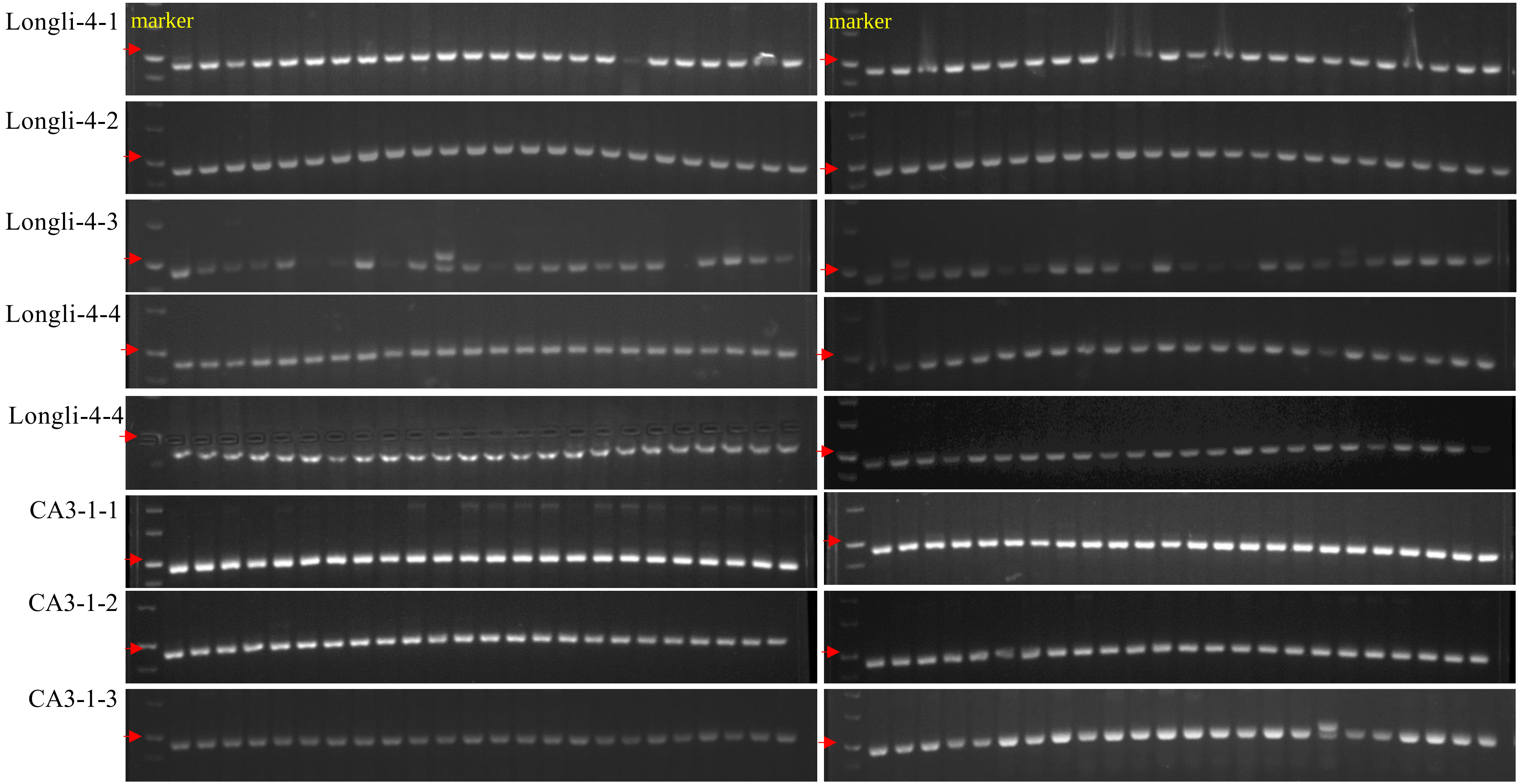

Chr15	Forward_primer	GTTGGATGTCACCATTGGAA
	Reverse_primer	TGCAAGCTAAGATAAGCTCACTACA

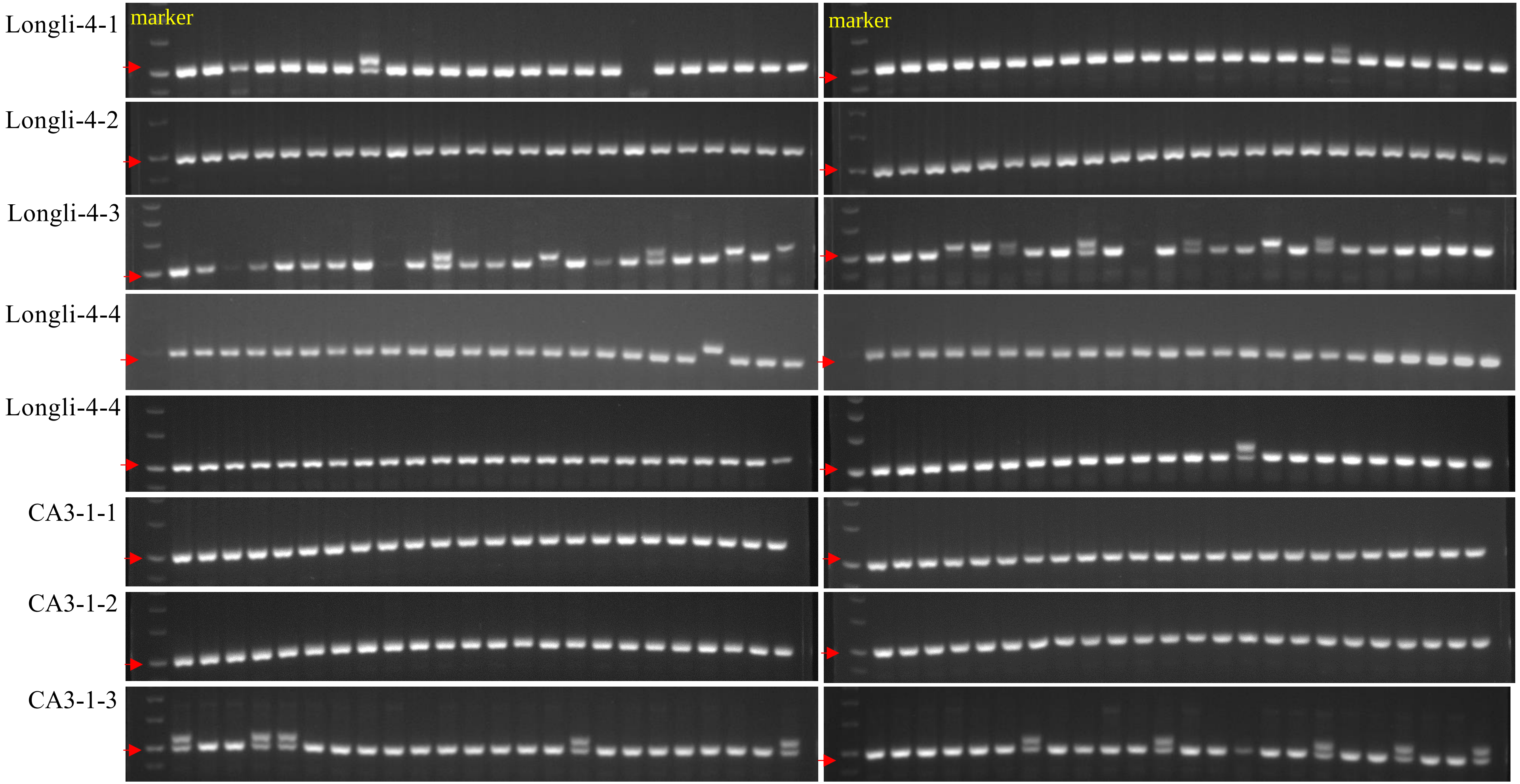

Chr16	Forward_primer	CCATGGACACTCTCAGGGTAA
	Reverse_primer	GCTTAGGATCGCAAAGCTGA

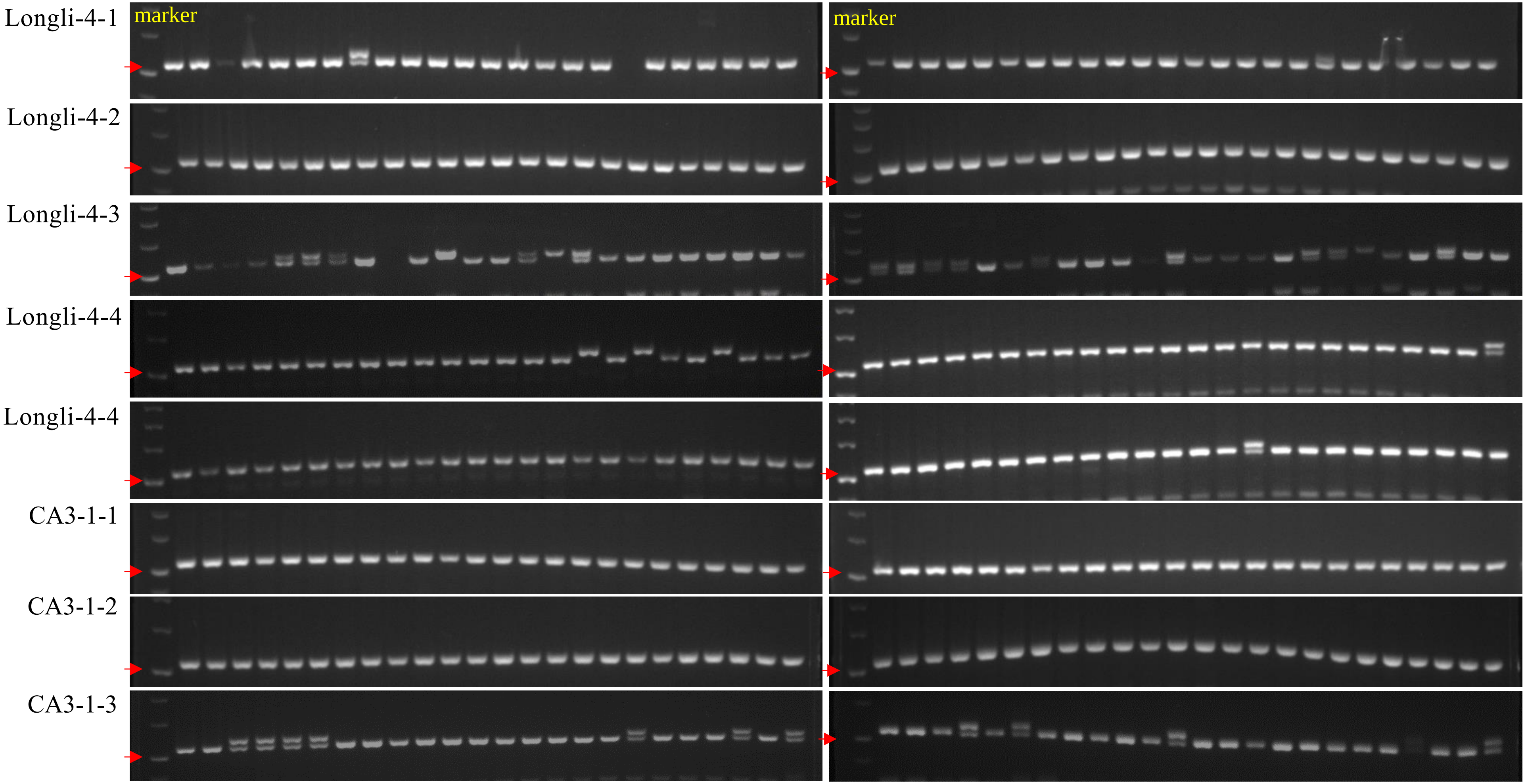

Chr17	Forward_primer	TTCCAGGATGGACTCTTTGG
	Reverse_primer	AACGTGTTCACACCATCACC

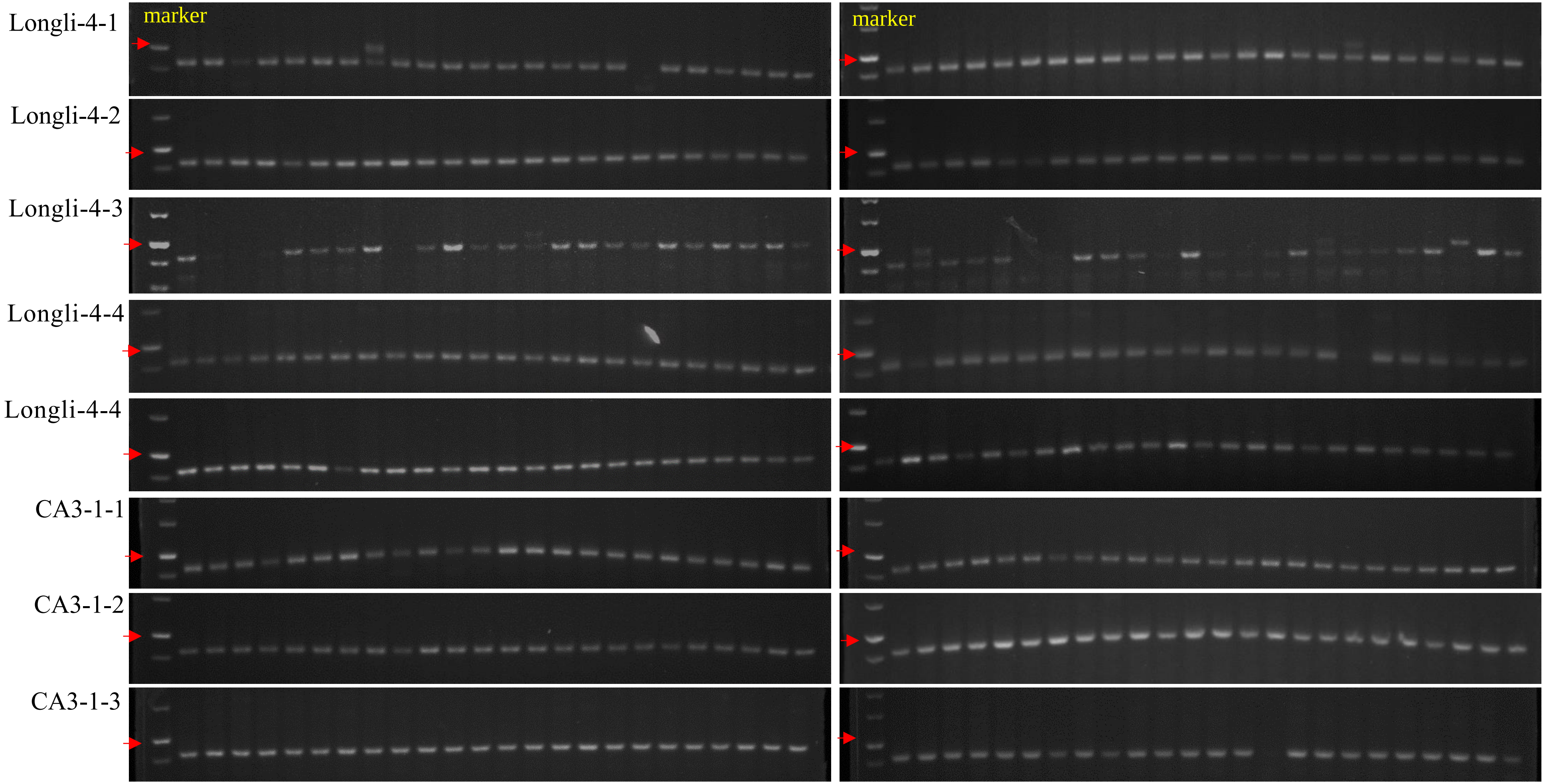

Supplement: Supplementary file 1 [file ijms-23-14030-s001.zip › Supplemental Figure S6 Genotyping using nine INDEL markers..pdf]
