# Supplementary material for: Genomic Variation Underlying the Breeding Selection of Quinoa Varieties Longli-4 and CA3-1 in China
Source: Int J Mol Sci. 2022 Nov 14;23(22):14030. doi: 10.3390/ijms232214030 (PMC9693436; doi:10.3390/ijms232214030)
Supplement: Supplementary file 1 [file ijms-23-14030-s001.zip › Supplemental Informations.pdf]

Supplemental Information

Supplemental Figures

Supplemental Figure S1 Phenotype comparisons of two quinoa varieties. **(A)** Four-month-old plants of Longli-4 and CA3-1. **(B-G)** Morphological comparisons of Longli-4 and CA3-1 plants, top **(B, E)**, middle **(C, F)**, and basal **(D, G)** stems. **(H)** Boxplots of plant height. **(I)** Stacked bar charts of internode length. Boxplots of lateral branch number **(J)**, and stem diameter **(K)** of Longli-4 and CA3-1. **(L-O)** Lamina **(L)**, top inflorescences from four individuals of each variety **(M)**, floret **(N)**, and immature grains **(O)**. **(P-R)** Leaf area **(P)**, panicle length **(Q)**, and panicle diameter **(R)** of Longli-4 and CA3-1.

Supplemental Figure S2 Stacked bar charts of variation numbers in each of Longli-4 and CA3-1. **(A-D)** The exact numbers of SNPs (**A, B**) and INDELs (**C, D**) are shown based on variation groups. (**E-H**) The exact numbers of SNPs (**E, F**) and INDELs (**G, H**) are shown based on annotation groups. **A, C, E, G,** Longli-4; **B, D, F, H**, CA3-1. The caption is the same as that in Figure 4 in main text. The A sub-genome of quinoa (red) includes Chr02, Chr04, Chr07, Chr08, Chr10, Chr12, Chr13, Chr14 and Chr15, while the B sub-genome (blue) includes Chr01, Chr03, Chr05, Chr06, Chr09, Chr11, Chr16, Chr17 and Chr18.

Supplemental Figure S3 Stacked bar charts of numbers of the pairwise SNPs and INDELs in Longli-4 and CA3-1. **(A, D)** Exact numbers of SNPs in Longli-4 (A) and CA3-1 (D). **(B, E)** Exact numbers of INDELs in Longli-4 (B) and CA3-1 (E). **(C, F)** SNPs (C) and INDELs (F) are classified into seven groups based on annotation information, and numbers of all groups on each chromosome are shown as a stacked bar. The caption is the same as that in Figure 6 in main text. The chromosomes from the A and B sub-genomes of quinoa are shown as those in Supplemental Figure S2.

Supplemental Figure S4 The shared SNPs and INDELs in Longli-4 and CA3-1. **(A)** Numbers of SNPs in six groups on each chromosome. **(B, D)** Numbers of SNPs (B) and INDELs (D) in seven annotation groups on each chromosome. **(C)** Numbers of INDELs in twelve groups on each chromosome. The caption is the same as that in Figure 7 in main text. The chromosomes from the A and B sub-genomes of quinoa are shown as those in Supplemental Figure S2.

Supplemental Figure S5 Boxplots of the heterozygosity values of SNP and INDEL in two sub-genomes. LL4S_A, the SNP of Longli-4 sub-genome A, LL4I_A, the INDEL of Longli-4 sub-genome A, LL4S_B, the SNP of Longli-4 sub-genome B, LL4I_B, the INDEL of Longli-4 sub-genome B, CA3-1S_A, the SNP of CA3-1 sub-genome A, CA3-1I_A, the INDEL of CA3-1 sub-genome A, CA3-1S_B, the SNP of CA3-1 sub-genome B, and CA3-1I_B, the INDEL of CA3-1 sub-genome B. The chromosomes from the A and B sub-genomes of quinoa are shown as those in Supplemental Figure S2.

Supplemental Figure S6 Genotyping using nine INDEL markers. For each INDEL marker, the chromosome ID and the forward and reverse primer sequences are shown in the top panel. The PCR products were detected using agarose gel electrophoresis and the DL500 ladder (Takara, Japan) was used to show the size of PCR products. The photography for each plant of each INDEL marker is shown in the bottom panel. Six plants from different sites with different cultivation methods were harvested and 48 progenies of each plant were used to test the outcrossing rates. For each plant, the genotypes of the progenies should be: AA and/or AB, the outcrossing rate was calculated as: [No. (AB) / No. (AB + AA)] ×100%. If the third genotype (BB) was obtained for one INDEL marker (e.g., Chr15, Chr16,

and Chr17 INDELs for Longli-4-3 progenies), the PCR result was assigned as Non-Applicable (NA). Ninety-six seedlings from the commercial grains of Longli-4-4 were used for genotyping and three genotypes AA, AB and/or BB were found. The outcrossing rate was calculated as: [No. (AB + BB) / No. (AB + AA+ BB)] ×100%.

Supplemental Tables

Supplemental Table S1 The distribution of SNPs or INDELs.

Supplemental Table S2 The non-synonymous and stop-gain genes caused by the variations in Longli-4, CA3-1 or both of them.
