# Supplementary figures and images for: Genomic Variation Underlying the Breeding Selection of Quinoa Varieties Longli-4 and CA3-1 in China
Source: Int J Mol Sci. 2022 Nov 14;23(22):14030. doi: 10.3390/ijms232214030 (PMC9693436; doi:10.3390/ijms232214030)

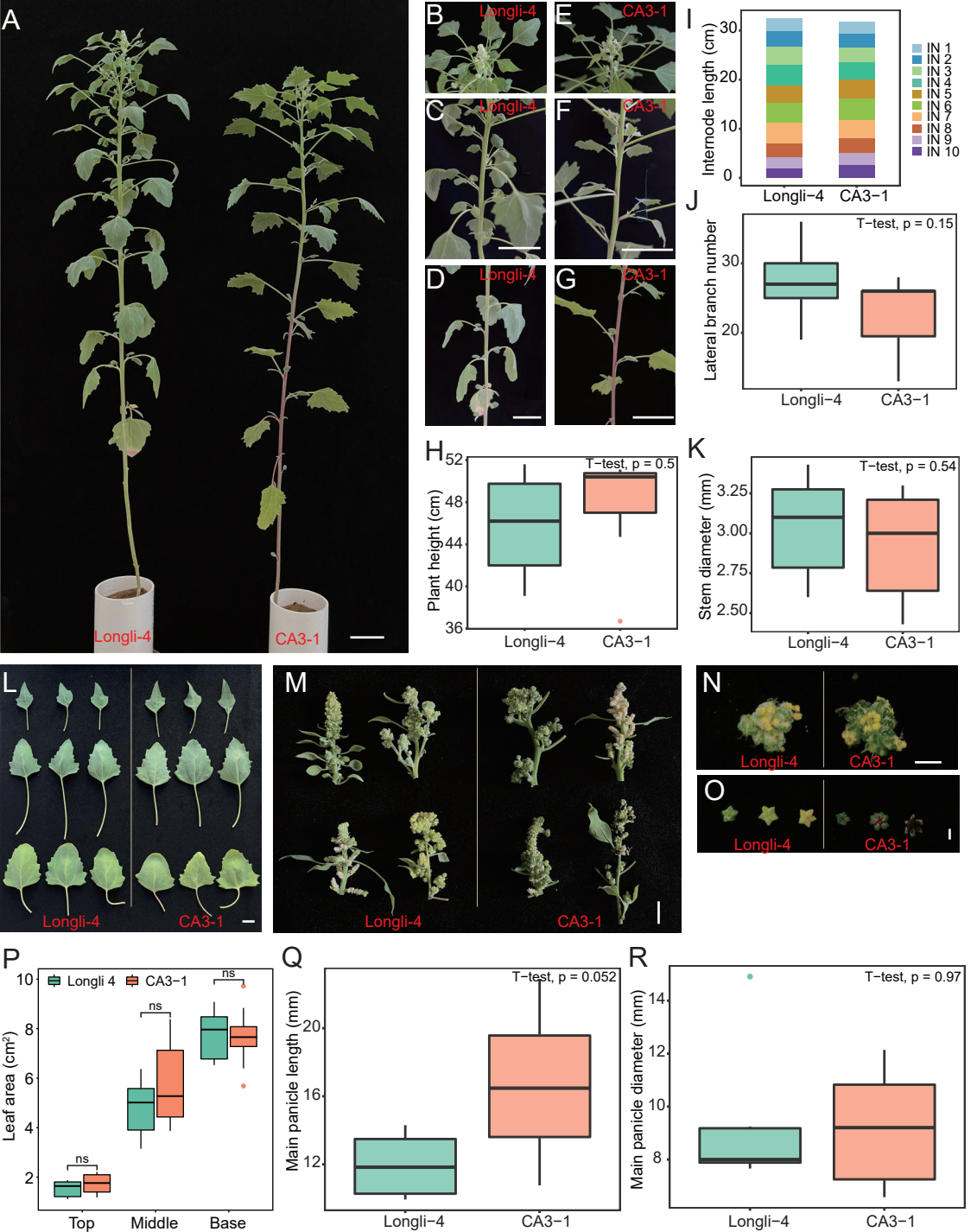

Supplement: Supplementary file 1 [file ijms-23-14030-s001.zip › Supplemental Figure S1 Phenotype comparisons of two quinoa varieties..pdf]

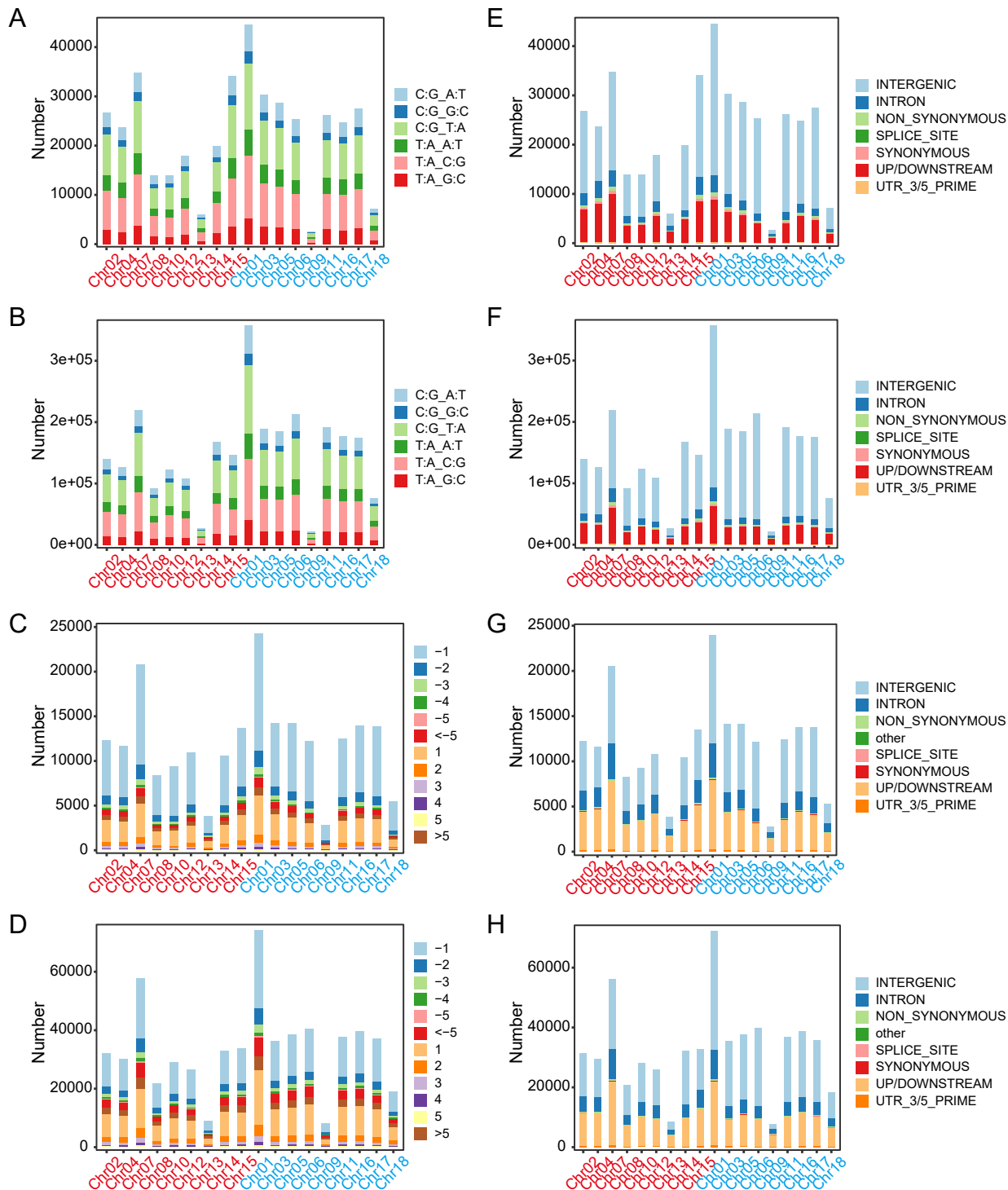

Supplement: Supplementary file 1 [file ijms-23-14030-s001.zip › Supplemental Figure S2 Stacked bar charts of variation numbers in each of Longli-4 and CA3-1..pdf]

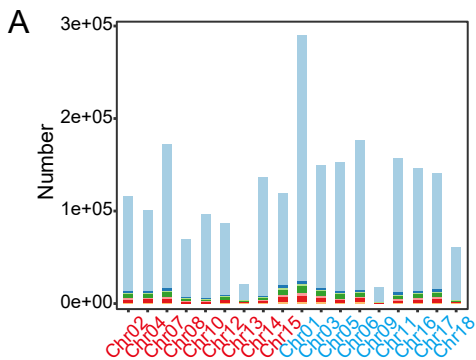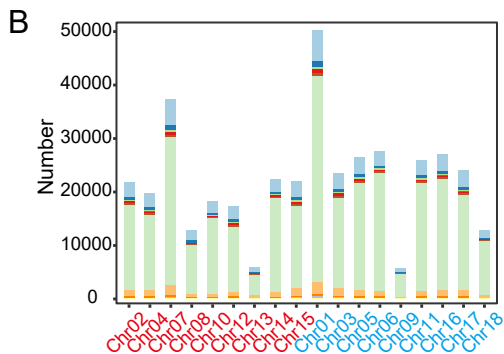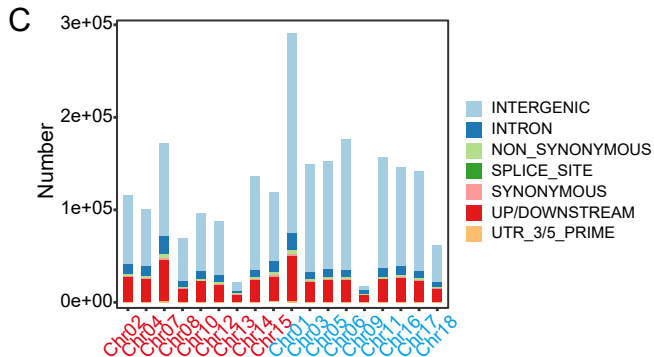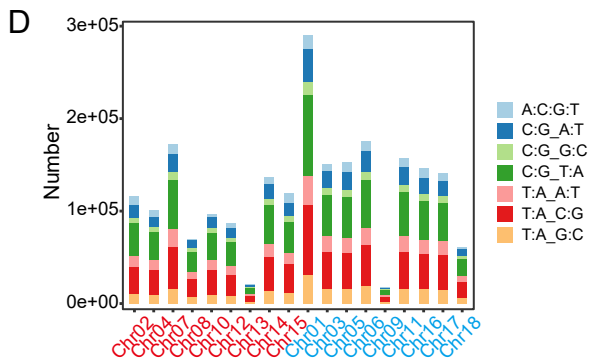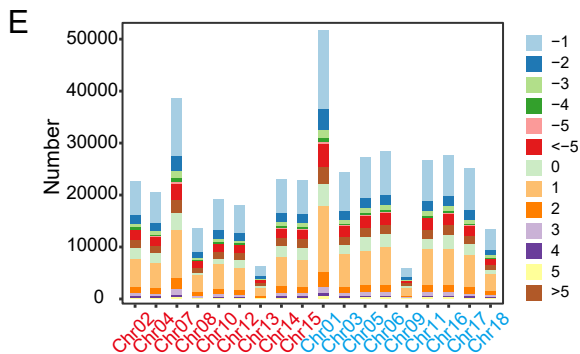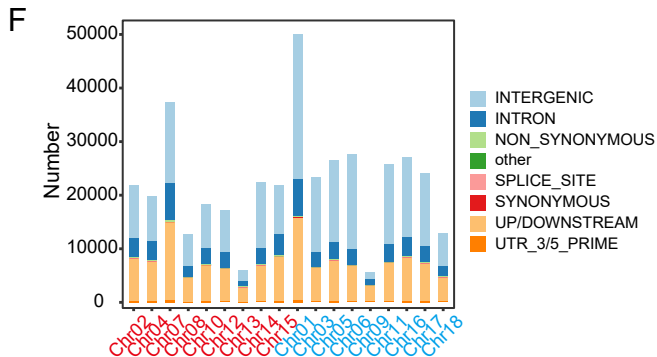

Supplement: Supplementary file 1 [file ijms-23-14030-s001.zip › Supplemental Figure S3 Stacked bar charts of numbers of the pairwise SNPs and INDELs in Longli-4 and CA3-1..pdf]

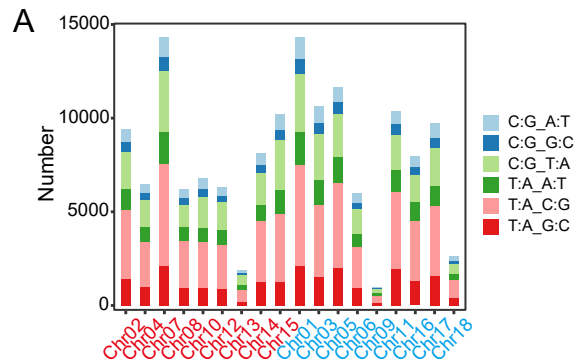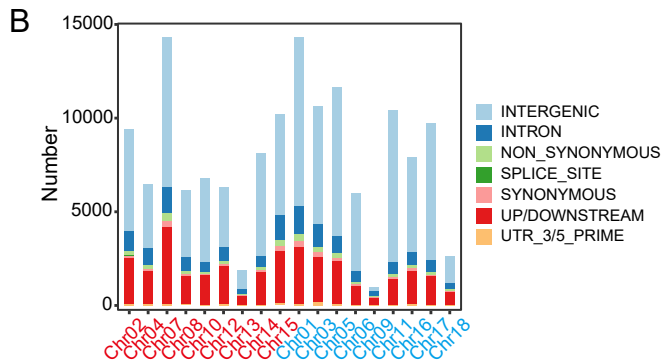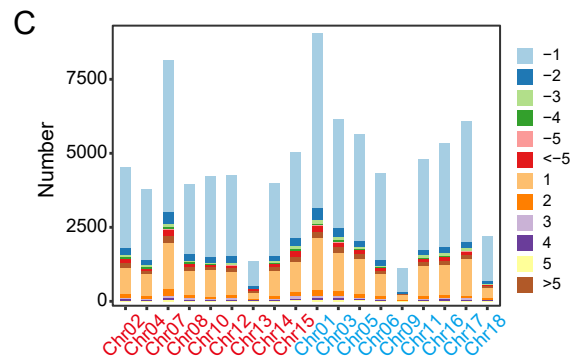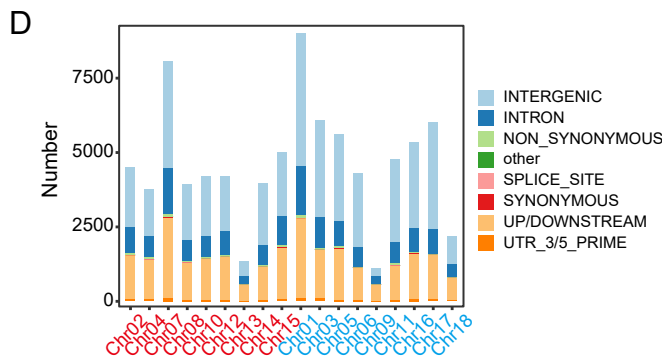

Supplement: Supplementary file 1 [file ijms-23-14030-s001.zip › Supplemental Figure S4 The shared SNPs and INDELs in Longli-4 and CA3-1..pdf]

**A**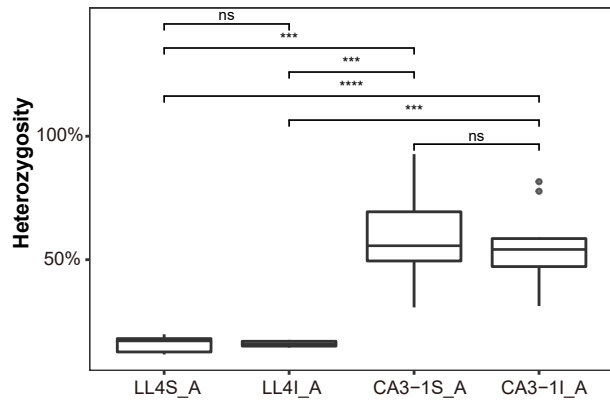**B**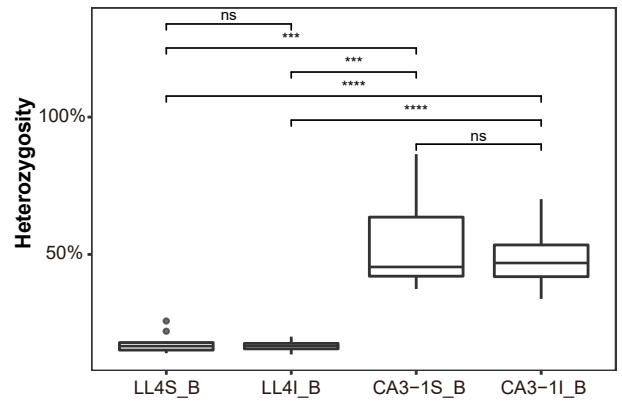

Supplement: Supplementary file 1 [file ijms-23-14030-s001.zip › Supplemental Figure S5 Boxplots of the heterozygosity values of SNP and INDEL in two sub-genomes..pdf]
